# Prognostic value of 5-microRNA based signature in T2-T3N0 colon cancer

**DOI:** 10.1007/s10585-016-9810-1

**Published:** 2016-08-02

**Authors:** Maciej Bobowicz, Marcin Skrzypski, Piotr Czapiewski, Michał Marczyk, Agnieszka Maciejewska, Michał Jankowski, Anna Szulgo-Paczkowska, Wojciech Zegarski, Ryszard Pawłowski, Joanna Polańska, Wojciech Biernat, Janusz Jaśkiewicz, Jacek Jassem

**Affiliations:** 1 Department of Surgical Oncology, Medical University of Gdansk, Gdansk, Poland; 2 Department of Oncology and Radiotherapy, Medical University of Gdansk, 7 Dębinki St., 80-211 Gdańsk, Poland; 3 Department of Pathomorfology, Medical University of Gdansk, Gdańsk, Poland; 4 Institute of Automatic Control, Data Mining Group, Silesian University of Technology, Gliwice, Poland; 5 Institute of Forensic Medicine, Medical University of Gdansk, Gdańsk, Poland; 6 Department and Clinic of Oncologic Surgery, Collegium Medicum, Nicolaus Copernicus University, Bydgoszcz, Poland; 7 Department of Clinical Oncology, Centre of Oncology, Bydgoszcz, Poland

**Keywords:** microRNA expression, colon cancer, metastasis, prognostic marker, miR-1300, miR-939

## Abstract

The role of adjuvant chemotherapy in stage T2-T3N0 colon cancer (CC) is controversial and there are currently no reliable factors allowing for individual selection of patients with high risk of relapse for such therapy. We searched for microRNA-based signature with prognostic significance in this group. We assessed by qRT-PCR expression of 754 microRNAs (miRNAs) in tumour samples from 85 stage pT2-3N0 CC patients treated with surgery alone. MiRNA expression was compared between two groups of patients: 40 and 45 patients who did and did not develop distant metastases after resection, respectively. Additionally, miRNA expression was compared between CC and normal colon mucosa samples and between the mismatch repair (MMR) competent and deficient tumours. Low expression of miR-1300 and miR-939 was significantly correlated with shorter distant metastasis-free survival (DMFS) in Cox univariate analysis (p.adjusted = 0.049). The expression signature of five miRNAs (miR-1296, miR-135b, miR-539, miR-572 and miR-185) was found to be prognostic [p = 1.28E−07, HR 8.4 (95 % CI: 3.81–18.52)] for DMFS and cross-validated in a leave-one-out analysis, with the sensitivity and specificity of 74 and 78 %, respectively. The expression of miR-592 was significantly associated with the MMR status (p.adjusted <0.01). The expression of several novel miRNAs were found to be tumour specific, e.g. miR-888, miR-523, miR-18b, miR-302a, miR-423-5p, miR-582-3p (p < 0.05). We developed a miRNA expression signature that may be predictive for the risk of distant relapse in early stage CC and confirmed previously reported association between miR-592 expression and MMR status.

**Electronic supplementary material:**

The online version of this article (doi:10.1007/s10585-016-9810-1) contains supplementary material, which is available to authorized users.

## Background

Colon cancer (CC) is the fourth greatest cause of cancer-related deaths worldwide [1]. Around 10–20 % of stage II patients (pT3/T4N0) will develop distant metastases after curative resection [2]. Currently, adjuvant chemotherapy is recommended in T4N0 patients with additional clinico-pathological adverse features [2]. The use of adjuvant chemotherapy in stage II CC patients results in the improvement of the 5-year survival by a mere 2–4 % [3, 4]. pT2N0 and pT3N0 patients are usually not prescribed adjuvant chemotherapy, even though a proportion of them will develop distant metastases. Hence, the identification of patients with high risk of dissemination in this subset may optimise the use of adjuvant therapies.

Molecular traits have been explored as a potential prognostication tool for CC. Mutations in oncogenes (e.g. *TP53, B*-*raf or* *K*-*ras*) have been shown to carry prognostic information, however they are insufficient for individual patient selection to adjuvant chemotherapy [5–9]. Microsatellite instability (MSI-H) has been repeatedly shown to correlate with CC prognosis [10, 11]. However MMR deficiency (dMMR) occurs in only around 15 % of sporadic CCs [12], and therefore remains irrelevant to the majority of CC cases.

Recently, prognostic gene-expression signatures have been developed in CC, among which Colon OncotypeDx [13] and the six-cluster gene expression ColoPrint [14] have been extensively validated in retrospective series. However, stage II–III CC patients identified by these classifiers as carrying ‘high risk’ have an approximately 60 % chance of 5-year survival with surgery alone. Therefore, a search for more specific prognostic classifiers is warranted.

MicroRNAs (miRNAs) are short, non-coding RNAs that play key roles in cancer cell [15]. These molecules regulate gene expression by binding to the target sequences of mRNA, which results in hindered translation [16]. It is estimated that miRNAs tune the expression of more than 30 % of human genes [17]. Notably, miRNA is stable in formalin-fixed paraffin-embedded (FFPE) samples, and therefore their analysis is not significantly affected by the storing time of tissue samples [18].

Recently, Zhang et al. [19] demonstrated the prognostic value of 5-miRNA-expression signature in stage II CC, and validated it in sizeable validation cohorts. A population-based translational study by Slattery et al. [20] confirmed the prognostic value of several other miRNAs. In another study, miR-362-3p was positively validated as prognostic in CC [21]. However, next generation sequencing-based study using fresh frozen material did not detect any prognostic miRNAs [22].

In the current study, we explored prognostic value of miRNA expression in stage T2-T3N0 CC, with the aim of developing a multi-miRNA prognostic expression signature. To this end, we assessed the expression of 754 miRNAs with qRT-PCR in FFPE samples from patients with or without distant relapse. Additionally, we investigated miRNA expression associations with microsatellite instability [23] and compared expression of miRNA between CC and normal colon mucosa (NCM) samples.

## Methods

### Patients

This study was approved by the Ethics Committee of the Medical University of Gdańsk. The study subjects were pT2-3N0 CC patients who underwent curative resection between 2001 and 2011, and either did or did not develop distant metastases. No-relapse group comprised patients with relapse-free survival of at least 4 years. Both groups were matched by major clinico-pathological features. Patients who developed isolated local or nodal recurrence were excluded. All patients underwent pathologically confirmed complete hemicolectomy or sigmoidectomy. To avoid confounding effect of occult nodal disease (active lymphatic spread), all cases were required to have had at least 12 lymph nodes excised. None of the patients received preoperative or postoperative chemotherapy.

### Pathological and molecular analyses

The study material was archival FFPE blocks containing primary tumour samples obtained at resection. All cases were reviewed using hematoxylin-eosin (HE) staining independently by two pathologists (PC and WB) to confirm CC diagnosis, in accordance with the WHO criteria. One slice from each block was HE stained and reviewed, and the block with the highest percentage of cancer tissue was chosen for molecular analysis. To further decrease the content of non-carcinomatous tissue, surrounding non-neoplastic component (normal mucosa, muscular layer, pericolic fat and necrotic tissue) was removed. The macrodissected blocks were required to obtain at least 80 % of viable tumour tissue. To avoid cross-contamination, the blades used for macrodissection and for slice cutting were changed after each case.

Four slices of 20 µm each were cut for total RNA isolation with RecoverAll Kit (Ambion). The concentration of RNA was assessed in NanoDrop^®^. Reverse transcription was carried out with 750 ng of RNA with TaqMan MiRNA RT kit (Applied Biosystems) and pools A and B of stem-loop primers (Megaplex™ Primer Pools, Human Pools Set v3.0, Applied Biosystems), in accordance with the manufacturer’s instructions. These pools contain primers specific for 377 different miRNAs, therefore to obtain cDNA for 754 miRNAs, in each sample reverse transcription was carried out twice—with pool A and pool B primers. cDNA was quantified by qRT-PCR, with the use of miRNAs specific primer pairs, fluorescent TaqMan probes and polymerase with 5′ nuclease activity, in microfluidic cards (TaqMan^®^ Array Microfluidic Cards, Applied Biosystems) in HT 7900 cycler (Applied Biosystems), with reaction conditions in accordance with manufacturer’s instructions (Applied Biosystems). Raw expression results (Ct values) were obtained through SDS.2.1 (Applied Biosystems) software.

All tumour slides were also assessed for expression of MMR proteins by immunohistochemistry (IHC) in tissue microarrays (TMAs) that included two cores of 1.5 mm diameter. After cutting 4 μm slides, the TMAs were stained in Dako autostainer for MLH1 (clone ES05, Dako, ready to use), MSH2 (G219-1129, Cell Marque, 1:200) and MSH6 (clone EP49, Dako, ready to use). The complete lack of IHC reaction for MLH1, MSH2 or MSH6 in cancer tissue, with retained expression in the surrounding stroma, was considered an indicator of microsatellite instability (MSI).

The NCM samples were obtained from the free surgical margins of the pathological specimen from which the CC sample was acquired.

### Statistical analysis

The primary clinical endpoint was distant metastasis-free survival (DMFS). The number of miRNAs that were expressed in at least 25, 50, 75 and 100 % of the samples in both analysed groups (‘relapsed’ or ‘non-relapsed’) were calculated. MiRNAs with no amplification signal (Ct ≥ 40) in less than 5 % of samples were included in the prognostic analyses. The rationale for this threshold for expression positivity is provided in the Supplementary material. Undetermined values of expressions (Ct ≥ 40) for this set of miRNAs were imputed by EM-based model of the missing data mechanism [24]. The individual Ct values for target miRNAs were normalised against the geometrical mean of the Ct values of U6 RNA, RNU44 and RNU48, and nine most stably expressed miRNAs that were determined with use of the NormFinder application (Appendix A, Table 1 in Supplementary material) [25]. Expression of miRNAs obtained with the 2^−(ΔCt)^ method [26, 27] was used to calculate fold change and its 95 % confidence interval. Expression of miRNAs obtained with the ΔCt method was used in subsequent analysis. Lilliefors test was used for the verification of the hypothesis on normality of the analysed signal. Univariate analyses were performed to examine the normalised miRNA expression changes between both patient groups with the use of non-parametric Mann–Whitney test and univariate Cox regression, with DMFS information included. P values were adjusted for multiple hypotheses testing using Benjamini-Hochberg algorithm. All statistical inferences were performed at significance level equal to 0.05. The DMFS curves were generated using Kaplan–Meier method. The clinical relevance of individual miRNAs was further tested in Cox multivariate models that included miRNA expression (as −∆Ct), the T stage and the histological grade. The negative ∆Ct allowed for the intuitive interpretation of resulting HR values, e.g. for the cases where the high miRNA expression was associated with the high risk of relapse, the obtained HR value was >1. The T feature was projected to a binary status (pT2 vs. pT3) and the grade was included with the values of 1, 2 or 3, respectively.

Multivariable logistic regression model was constructed to find an expression signature that describes the dependence between miRNA expression and recurrence status. For each patient recurrence score (RS) was estimated as prediction of logistic regression model. Given the large number of potential explanatory variables, regression model was limited to variables that were associated with p values ≤ 0.2 in the univariate analyses [28]. The number of model predictors was found using forward selection scheme, with manual tuning based on Bayesian information criterion, R^2^ and a p value of likelihood ratio test indicating difference from a smaller model. Contribution of an individual predictor was measured using the Wald test. Optimal threshold for RS was found by maximizing positive predictive value (PPV) with the constraint on negative predictive value (NPV) higher than 0.8. Additionally, regularised parameters of estimated model were obtained by using LASSO regression [29]. Prognostic capabilities of the obtained model were checked by internal leave-one-out cross-validation. The clinical relevance of the signature was further tested in Cox multivariate models that included T stage and histological grade. The study including 85 patients, comprising 40 patients with cancer recurrence—‘events’, and 45 without disease recurrence ‘controls’, had 80 % power to detect a difference in survival between the ‘high’ and the ‘low’ risk groups at the level of HR = 1.81 with 0.05 alpha value.

The gene expression omnibus (GEO) data repository contains the biological experiment (GSE63119) that could be used to confirm the expression of miRNAs constituting the prognostic signature. In GSE63119 series it was possible to examine miRNA expression values (obtained by Illumina sequencing) of 50 CC patients with and without metachronous metastases [30]. In this paper, reads for all isoforms of a given miRNA were counted and log2 transformed as a basis for miRNA expression estimation. Indirect validation of prognostic value of the expression of miRNAs from the signature was carried out through hierarchical clustering of original and external dataset, and by building logistic regression model on external data set.

To find miRNAs that were expressed only in CC or NCM samples, we discretised the raw Ct values to the following expression categories: (1) *no amplification signal*: Ct ≥ 35, (2) *expression*: Ct < 35. Discretised, nominal scale data derived from paired samples of NCM and CC were analysed by the McNemar test. For the comparison of miRNA expression between CC and NCM Ct values for target miRNAs were normalised according to the proposed method described in the preceding section. The expression of miRNAs obtained with the ΔCt method was analysed using the paired sample *t* test. Applying a condition on miRNA to be significantly differentiating between CC and NCM samples in both the original and validation experiments is very conservative, especially in case of small sample sizes. As an alternative the selection algorithm substitutes set of p values by one p value, named combined p value. Combined p value was calculated using the weighted Z test that requires one-sided test p values [31]. When finding one-sided p values for independent experiment, we could directly include the information about the required increase/decrease of miRNA expression observed in original dataset.

ArrayExpress repository contains the data from a biological experiment that could be used to validate miRNAs found to have an amplification signal only in NCM or CC. In E-GEOD-46,622 experiment authors examined miRNA expression values by Illumina sequencing of matched benign, primary tumour and metastatic tissues of eight colorectal cancer patients [32]. To obtain a measure of miRNA expression, reads on target regions were counted and log2 transformed. Due to the high rate of false positive results in the low range of expression for sequencing experiments, we analysed the distribution of log2 signal using Gaussian mixture model and k-means algorithm and found the threshold value for expression signal [33]. The values of log2 read counts lower than the estimated threshold were considered as having no amplification signal.

## Results

A total of 85 patients were studied, including 40 who did and 45 who did not develop distant metastases for at least 4 years, with a median follow-up of 3.9 and 5.8 years, respectively. The sites of metastases included lungs, liver, bones and skin. Major clinical characteristics in relapsed and non-relapsed groups did not differ significantly, except for stage (more stage I patients in the control group; Table 1). Out of 754 analysed miRNAs, 159 (21 %) with Ct < 40 of 0–5 % were considered expression negative. A total of 465 miRNAs (62 %) were expressed in at least 25 % of all samples, 398 miRNAs (53 %) in at least 50 % of samples, 340 miRNAs (45 %) in at least 75 % of samples and 99 miRNAs (13 %) in all samples. Expression of 229 miRNAs (30 %) was present in more than 95 % of samples. These miRNAs were subjected to the process of imputation and further analysed. The RNA quality was typical of FFPE samples and the Ct values for RNAs chosen for normalisation are shown in Appendix A, Table 1 in Supplementary material.

**Table 1:** Patients’ clinic-pathological characteristics

Variable	Category	All patients (N = 85)	Relapsed group (N = 40)	Non-relapsed group (N = 45)	p
Sex	Male	50	26	24	0.275
	Female	35	14	21	
Stage	I	40	12	28	0.003
	IIA	45	28	17	
Grade	G1	11	3	8	0.187
	G2	70	34	36	
	G3	2	1	1	
Mean age (range)	Years	66 (32–87)	67 (32–83)	65 (42–87)	0.92
Median follow-up (range)	Years	3.17 (0.5–11.9)	1.7 (0.5–3.08)	4.25 (4.0–11.9)	8.97e−14
MMR status	Deficient/total	13/85	6/40	7/45	0.94

MMR - DNA mismatch repair system

### MiRNA expression and distant metastasis free survival

In a univariate Cox regression model, there was a statistically significant association between low expression of miR-1300 and miR-939 and a shorter DMFS (after correction for multiple comparisons, p = 0.049). In the multivariate Cox prognostic models including miRNA expression, pT stage and tumour grade, low expression of miR-939 [p = 0.011, HR 1.53 (95 % CI 1.10–2.12)] and pT stage [p = 0.05, HR 2.68 (95 % CI 1.00–7.19)] were independently correlated with DMFS. The comparison of miRNAs median expression between the groups of patients with and without disease recurrence did not result in significant differences after correction for multiple testing. In order to identify miRNAs potentially involved in metastases formation, we searched for miRNAs with expression that was simultaneously correlated with DMFS (unadjusted Cox test p < 0.05) and was different between the patient groups with and without disease recurrence (unadjusted U test p < 0.05). The expression of miR-1300, miR-939, miR-596, miR-572, miR-210, miR-1303, miR-422a, miR-1260, miR-7#, miR-1296, miR-185, miR-26a-1*, miR-639, miR-650 and miR-155 met both criteria (Table 2). The miRNAs with prognostic or potentially prognostic significance in CC were mostly downregulated in the group with disease recurrence, with the exception of miR-1296 that was upregulated. The full list of correlations of miRNA expression with DMFS (Cox test) and comparisons of miRNAs expression between the relapsed and non-relapsed groups (U test) are shown in Appendix A, Table 2 in Supplementary material.

**Table 2:** MiRNAs expression differences between the groups with and without colon cancer relapse

miRNA name	Non-meta ΔCt		Meta ΔCt		Fold	FC	FC	U test		Cox regression				
	Mean	SD	Mean	SD	Change	Low CI	High CI	P value	FDR	P value	FDR	HR	HR 95 % CIs	
hsa-miR-1300	7.44	1.14	8.32	2.00	0.54	0.33	0.90	0.035	0.634	<0.001	0.044	1.411	1.173	1.697
hsa-miR-939	5.27	1.28	6.28	1.84	0.50	0.30	0.81	0.014	0.549	<0.001	0.044	1.394	1.158	1.678
hsa-miR-596	2.57	1.34	3.55	1.84	0.51	0.31	0.83	0.010	0.549	0.001	0.062	1.341	1.122	1.602
hsa-miR-572 (syg)	8.52	1.39	9.65	1.91	0.45	0.27	0.76	0.002	0.361	0.001	0.062	1.252	1.092	1.436
hsa-miR-210	3.37	1.20	4.01	1.14	0.64	0.45	0.92	0.012	0.549	0.012	0.306	1.380	1.073	1.774
hsa-miR-1303	7.80	1.30	8.73	1.93	0.52	0.32	0.87	0.040	0.634	0.013	0.306	1.222	1.043	1.433
hsa-miR-422a	5.34	1.68	6.33	2.20	0.51	0.28	0.92	0.032	0.634	0.014	0.306	1.207	1.039	1.403
hsa-miR-7#	6.37	1.55	7.11	1.58	0.60	0.37	0.97	0.037	0.634	0.021	0.306	1.219	1.030	1.442
hsa-miR-1296 (syg)	9.90	1.69	9.08	1.49	1.76	1.09	2.86	0.027	0.634	0.022	0.306	0.772	0.618	0.964
hsa-miR-185 (syg)	7.20	0.93	7.70	1.24	0.70	0.50	0.99	0.082	0.634	0.024	0.306	1.343	1.040	1.735
hsa-miR-650	6.76	1.33	7.39	1.44	0.64	0.42	0.98	0.049	0.634	0.029	0.306	1.273	1.024	1.582
has-miR-155	2.37	1.33	3.06	1.05	0.62	0.43	0.89	0.014	0.407	0.033	0.306	1.294	1.021	1.641
hsa-miR-539 (syg)	7.31	1.99	6.53	1.24	1.71	1.05	2.81	0.212	0.699	0.062	0.393	0.819	0.664	1.010
hsa-miR-135b (syg)	5.61	1.86	5.10	1.53	1.42	0.85	2.37	0.202	0.699	0.228	0.670	0.888	0.731	1.078

*Non-meta* the group that did not develop relapse, *Meta* the group that developed distant metastases, *ΔCt* Ct—geometric mean of 12 Ct normalisers, *FC* fold change, *FDR* false discovery rate, *HR* hazard ratio, *CI* confidence intervals, *syg* miRNAs constituting the 5-miRNA signature, SD standard deviation

**Fig. 1:**
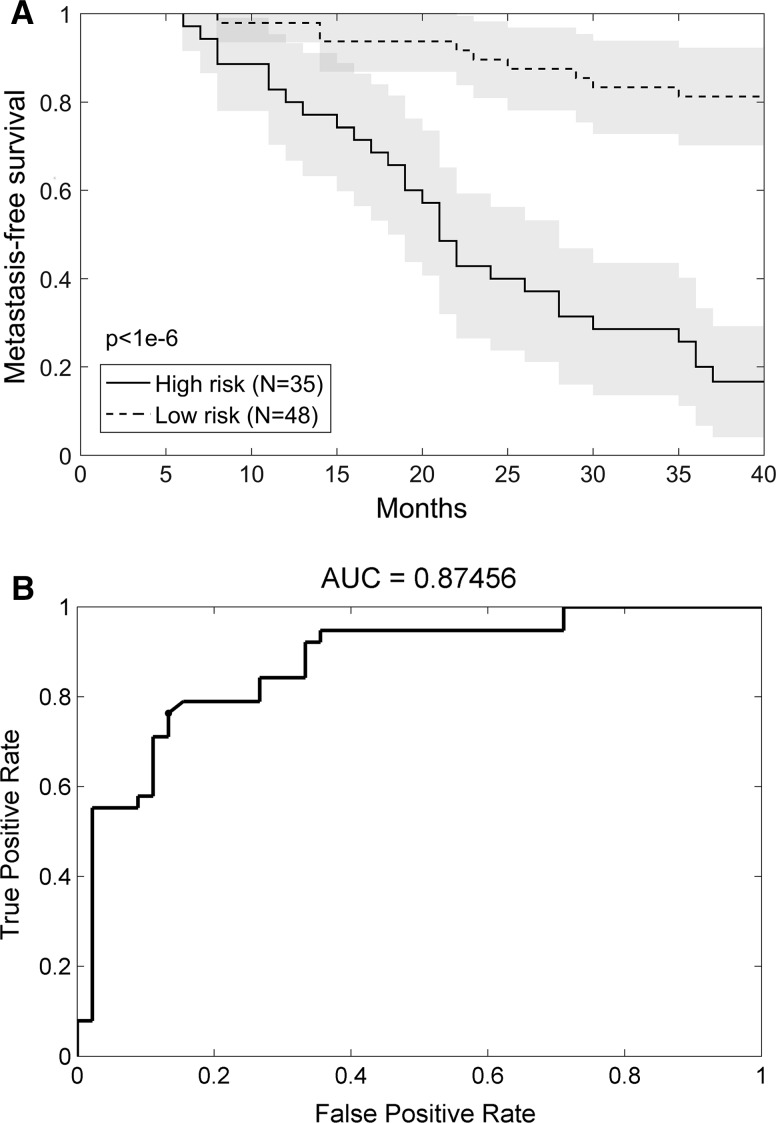
Metastasis-free survival according to the 5-miRNA expression signature (RS threshold = 0.5) (**a**) and the area under the curve for estimating 3 year metastasis-free survival using the 5-miRNA expression signature (**b**)

An algorithm for prognostic expression model generation resulted in an expression signature including five miRNAs (5-miRNA signature) that were either upregulated (miR-1296, miR-135b and miR539) or downregulated (miR-572 and miR-185) in the CC cases with subsequent relapse. The individual miRNA markers contributed to the signature with the following weights: logit(RS) = −3.61 − 0.72 × miR-135b + 1.45 × miR-185 − 0.59 × miR-539 − 0.61 × miR-1296 + 0.68 × miR-572. This signature’s risk score was strongly associated with DMFS [HR 8.4 (95 % CI: 3.81–18.52); p < 0.004], with sensitivity and specificity of 76 and 87 %, respectively (Fig. 1). The median DMFS for the ‘high-risk’ group was 21 months, and was not reached for the ‘low-risk’ prognostic group. The 3-year DMFS for the ‘high-risk’ and the ‘low-risk’ prognostic groups was 20 and 81 %, respectively. The NPV and PPV were 82 and 81 %, respectively, and the measure of the model being better than a chance was p = 6.28e^−08^ (χ^2^ test). In the multivariate Cox model, including the risk score, grade (combined grade 1 and 2 vs. grade 3) and pathological stage (pT2 vs. pT3), the risk score was the only variable that significantly correlated with DMFS [HR 8.91 (95 % CI 3.69–21.48]. Further, the miRNA expression signature was cross-validated in a leave-one-out analysis, yielding sensitivity and specificity of 74 and 78 %, respectively. The risk score was also correlated with the overall survival [HR 4.82 (95 % CI: 2.15–10.77); p = 1.24E^−04^].

### Indirect independent validation of the 5-miRNA prognostic signature

**Fig. 2:**
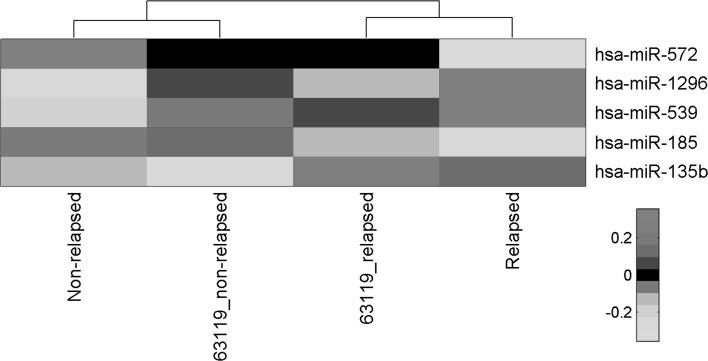
Results of hierarchical clustering of average expression data for miRNAs constituting the 5-miRNA prognostic signature in two independent experiments: the original data set and GSE63119 data set. Expression values measured in two different platforms were integrated using the z-score transformation. The clustering was performed on average signal from ‘relapsed’ and ‘non-relapsed’ samples in both experiments with Euclidean distance metric

All miRNAs constituting the 5-miRNA signature were found to be expressed in the external miRNA expression dataset (GSE63119) [30]. Hierarchical clustering using averaged expression signal for each of the five miRNAs from the signature correctly grouped the samples according to their metastasis status in both original and validation experiments, even though the average expression of miR-1296 and miR-572 showed some discordances between both datasets (Fig. 2). Expression value of five miRNAs in GSE63119 experiment was obtained in a different high-throughput platform than in the original set, and thus it was not possible to apply directly the 5-miRNA signature with the estimated model coefficients. However, by performing model building with the same rules that were applied to the original data set, we obtained a model that could distinguish relapsed and non-relapsed patients in the independent data set. Except for miR-1296, all miRNAs showed the pattern of association of expression signal (upregulation or downregulation) with metastasis status as in the original model. Created model was associated with metastasis status (AUC = 0.673) with sensitivity and specificity of 96 and 27 %, respectively. The NPV and PPV were 88 and 55 %, respectively.

### MiRNA expression according to tumour grade, stage and MIS (microsatellite instability) status

Out of 14 miRNAs with different expression according to histological tumour grade (p.unadjusted <0.05, U test; Appendix A, Table 3 in Supplementary material), only miR-124 and miR-1243 had higher expression in grade 2 or 3 versus grade 1 tumours. There were 21 miRNAs with expression lower in pT3 versus pT2 tumours (p.unadjusted <0.05, U test; Appendix A, Table 3 in Supplementary material). However, none of these differences reached significance after correction for multiple testing.

MMR-deficiency phenotype (dMMR) was present in 13 cases (15 %), and the remaining 72 cases (85 %) were MMR-competent (cMMR). The frequency of distant metastases in these groups was 53 and 58 %, respectively (p = 0.75). Expression of miR-592 was significantly higher in cMMR compared to dMMR CC with the fold change of 11.8× (U p.adjusted = 0.0021).

### MiRNA expression in colon cancer versus normal colon mucosa

**Fig. 3:**
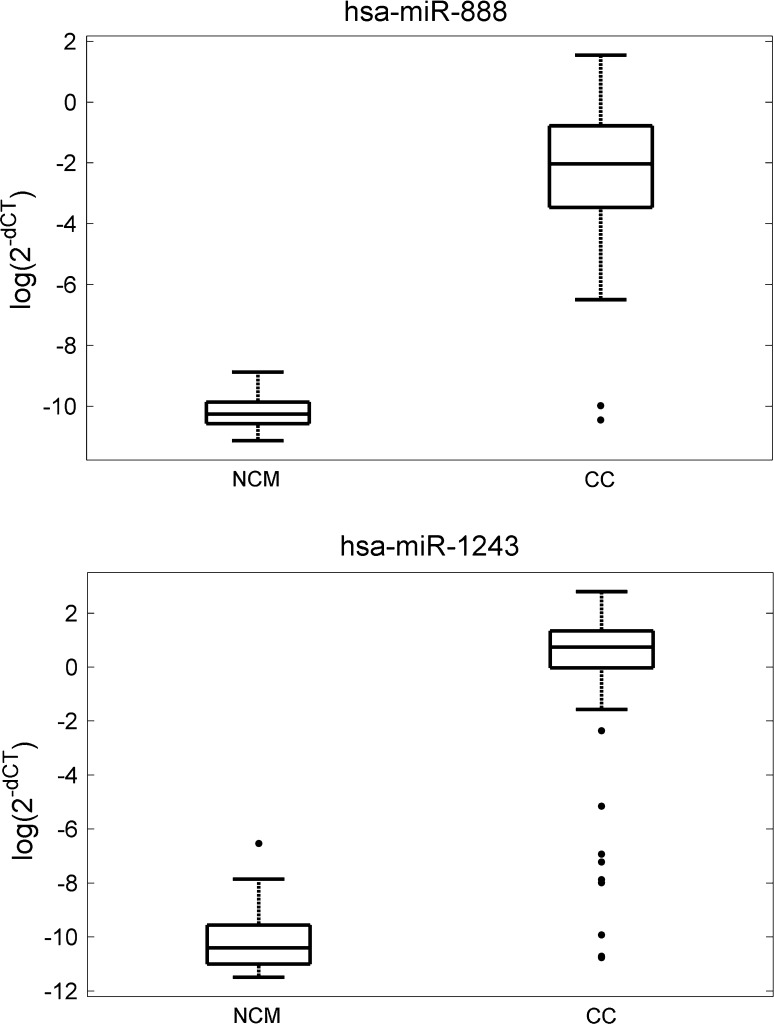
Expression of miR-888 and miR-1243 in normal colon mucosa (NCM) and in colon cancer (CC)

MiRNA expression was compared between the CC and NCM sample pairs from 14 CC patients. By analysing discretised data we found the expression of 30 miRNAs present specifically in primary tumours, e.g. miR-888, miR-523, miR-18b, miR-302a, miR-339-5p, miR-423-5p, miR-582-3p, miR-1243 (p < 0.05) and the expression of miR-299-5p and miR-1262 specific to NCM (p < 0.05) (Fig. 3). In the independent dataset (E-GEOD-46622) we found reads for 18 out of 32 miRNAs (56 %) differently expressed between CC and NCM in the original data set [32]. Eight reads for a given miRNA was a threshold for the expression signal positivity that corresponded to the threshold value of 3.048 in log2 scale found by using mixture model. By combining p values we found eight miRNAs significantly differentially expressed between CC and NCM common for both experiments, among which seven were tumour specific, i.e. miR-181c, miR-182, miR-32, miR-577, miR-301b, miR-656, miR-372 (p < 0.05) and miR-1262 was expressed specifically in NCM (p < 0.05).

By applying paired *t* test on ΔCt values we found 282 miRNAs that discriminate NCM and CC samples, among which 112 miRNAs with higher expression in CC samples, e.g. miR-888, miR-21, miR-582-3p, miR-523, miR-520c-3p (p < 0.05) and the remaining miRNAs with higher expression in NCM samples, e.g. miR-1262, miR-190b, miR-299-5p, miR-593, miR-633 (p < 0.05). In the independent dataset (E-GEOD-46622) we found reads for 77 miRNAs (27 %) from 282 discriminating CC and NCM samples. Among them there were five miRNAs that discriminated CC and NCM with the same profile of expression (increase/decrease) as in the original data set, i.e. miR-31, miR-192, miR-21, miR-145, miR-375 (p < 0.05). By combining p values we found 63 miRNAs significantly discriminating between CC and NCM common for both experiments, among which 30 were specific for tumours, e.g. miR-21, miR-31, miR-106b, miR-17, miR-135b (p < 0.05) and 33 were specific for NCM, e.g. miR-1262, miR-190b, miR-598, miR-195, miR-145 (p < 0.05).

## Discussion

We report here a 5-miRNA expression signature with potentially strong prognostic impact in early stage CC. To increase the robustness of our data, the analyses were limited to homogeneous groups of patients (pT2N0 and pT3N0 primaries) who underwent adequate radical surgery and had full follow-up data for at least 4 years. The study findings were corroborated in silico in an independent set of CC samples with annotated clinical follow-up information [30]. The signature is based on solid normalisation strategy that can be a reference for future prognostic studies.

The function of miRNAs constituting the signature is being increasingly recognised. Valeri et al. [34] previously reported negative prognostic significance of miR-135b overexpression in CC. Several oncogenic pathways (e.g. *APC/Wnt/β*-*catenin* and *PI3KCA*) converge on miR-135b, causing its progressive upregulation [34]. MiR-1296 was found to downregulate tumour suppressor *XAF1* in immortalised lymphoblastoid cells [35]. Interestingly, miR-539, upregulated in the ‘high-risk’ early stage CC, targets O-GlcNAcase (OGA), an enzyme that posttranslationally removes O-linked β-N-acetylglucosamines residues [36]. In vitro, O-GlcNAcylation enhanced the anchorage-independent growth and invasion of lung and CC cells [37], which is line with our findings. MiR-185 has been associated with tumour suppression function, whereby its low expression confers higher risk of dissemination [38, 39]. We have demonstrated for the first time in CC an inverse correlation between miR-939 expression and DMFS. The prognostic value of this miRNA was previously reported in stage I squamous cell lung cancer patients [40]. In hepatocytes miR-939 was shown to downregulate *human inducible nitric oxide synthase* (*hiNOS*), in a check-and-balance system that protects against undue consequences of excessive hiNOS activity [41]. On the other hand, in CC hiNOS activates Wnt/β-catenin pathway by negative regulation of *DKK1* [42]. Adverse prognostic impact of miR-939 downregulation may be therefore linked to increased nitric oxide synthesis that promotes metastasis formation by boosting Wnt/β-catenin signalling.

Our study confirms previously reported association between CC MMR status and miR-592 expression [43]. This concordance between two independent studies indicates their methodological accuracy and underscores important differences in epigenetics of cMMR and dMMR CC. Several novel miRNAs with expression differentiating CC from NCM were identified, including miR-888, miR-523, miR-18b, miR-302a, miR-423-5p, miR-582-3p and miR-299-5p, and the previously reported tumour specific expression of miR-181c, miR-182, miR-301b, miR-92a-1# confirmed [19, 44, 45].

The relevance of the above findings is further supported by the use of highly sensitive RT-PCR technique, suitable for capturing subtle differences in miRNA expression that are often undetectable with other platforms, e.g. the next generation sequencing (NGS) [46]. MiRNA-specific reverse transcription and target specific fluorescent probes allowed a high level of specificity of the expression signal. Among limitations of this study is the fact that RT-PCR requires a prior knowledge of sequence to be amplified and therefore does not allow for the discovery of new miRNAs or new variants of known miRNAs [47]. On the other hand, a detection bias of NGS towards miRNAs with uracil-rich sequences and a detection bias of oligonucleotide microarrays towards miRNAs with guanine-rich sequences was recently reported [48]. This study does not address the question of tumour microenvironment or cellular compartment localization of miRNA expression, which can be addressed by the use of in situ hybridization with locked nucleic acid (LNA) probes [49]. Still, in reference to RT-qPCR, in situ hybridization is more affected by variations in tissue procurement and fixation procedures [50].

Although this study includes homogeneous and carefully selected group of patients, its limitation is a relatively small sample size and an imbalance between ‘relapsed’ and ‘non-relapsed’ groups regarding pT stage. Hence, multi-institutional independent cohort validation of these results, preferably in the context of prospective study, is warranted to verify clinical utility of our findings.

## Electronic supplementary material

Below is the link to the electronic supplementary material.

Supplementary material 1 (DOCX 217 kb)

Supplementary material 2 (DOCX 192 kb)
