# Supplementary material for: Prognostic value of 5-microRNA based signature in T2-T3N0 colon cancer
Source: Clin Exp Metastasis. 2016 Aug 2;33(8):765–73. doi: 10.1007/s10585-016-9810-1 (PMC5110606; doi:10.1007/s10585-016-9810-1)
Supplement: Supplementary file 1 — Supplementary material 1 (DOCX 217 kb) [file 10585_2016_9810_MOESM1_ESM.docx]

Table 1. Normalization miRNAs for reverse transcription in pool A and pool B.

| **Primer**  **Pool** | **CT ≤25** | | | | **CT 26-30** | | | | **CT >30** | | | |
| --- | --- | --- | --- | --- | --- | --- | --- | --- | --- | --- | --- | --- |
| **A** | **miRNA** | **Average Ct** | **Variance of Ct** | **Coefficient of**  **Variation**  **of 2^-ΔCt** | **miRNA** | **Average Ct** | **Variance of Ct** | **Coefficient of**  **Variation**  **of 2^-ΔCt** | **miRNA** | **Average Ct** | **Variance of Ct** | **Coefficient of**  **Variation**  **of 2^-ΔCt** |
|  | miR-24 | 22.9 | 0.933 | 0.546 | miR-26a | 26.7 | 1.305 | 0.603 | miR-152 | 30.3 | 1.444 | 0.626 |
|  | miR-126 | 23.4 | 1.162 | 0.558 | let-7g | 27.6 | 1.268 | 0.585 | miR-324-3p | 30.5 | 1.340 | 0.548 |
|  | miR-200c | 24.1 | 1.055 | 0.599 | miR-27a | 28.3 | 1.193 | 0.774 | miR-197 | 31.5 | 1.517 | 0.646 |
|  | 9 normalising miRNAs  + 3 control panel RNAs |  |  |  |  | 26.4 | 0.999 | 0.502 |  |  |  |  |
| **B** | miR-1274B | 18.7 | 1.028 | 0.617 | miR-1825 | 27.4 | 1.195 | 0.627 | miR-30e-3p | 30.1 | 1.117 | 0.616 |
|  | miR-720 | 19.7 | 0.752 | 0.556 | miR-93# | 28.6 | 0.801 | 0.501 | miR-1285 | 31.3 | 1.010 | 0.632 |
|  | miR-1274A | 22.6 | 1.496 | 0.816 | miR-664 | 28.2 | 0.665 | 0.532 | miR-335# | 32.3 | 2.967 | 0.839 |
|  | 9 normalising miRNAs  + 3 control panel RNAs |  |  |  |  | 26.1 | 0.738 | 0.514 |  |  |  |  |

Table 2. MiRNAs expression in the ‘relapsed’ and ‘non-relapsed’ groups

| **miRNA name** | **Fold  change** | **FC  low CI** | **FC  high CI** | **U test** | | | **Cox regression** | | | | | |
| --- | --- | --- | --- | --- | --- | --- | --- | --- | --- | --- | --- | --- |
|  |  |  |  | **P-value** | **Q-value** | **FDR** | **P-value** | **Q-value** | **FDR** | **HR** | **HR 95% CI** | |
| hsa-miR-1300 | 0.54 | 0.33 | 0.90 | 3.51E-02 | 7.58E-01 | 6.34E-01 | 2.54E-04 | 4.92E-02 | 4.39E-02 | 1.41 | 1.17 | 1.70 |
| hsa-miR-939 | 0.50 | 0.30 | 0.81 | 1.38E-02 | 6.68E-01 | 5.49E-01 | 4.53E-04 | 4.39E-02 | 4.39E-02 | 1.39 | 1.16 | 1.68 |
| hsa-miR-596 | 0.51 | 0.31 | 0.83 | 1.04E-02 | 1.00E+00 | 5.49E-01 | 1.24E-03 | 8.00E-02 | 6.18E-02 | 1.34 | 1.12 | 1.60 |
| hsa-miR-572 | 0.45 | 0.27 | 0.76 | 1.86E-03 | 3.61E-01 | 3.61E-01 | 1.28E-03 | 6.18E-02 | 6.18E-02 | 1.25 | 1.09 | 1.44 |
| hsa-miR-210 | 0.64 | 0.45 | 0.92 | 1.18E-02 | 7.64E-01 | 5.49E-01 | 1.20E-02 | 4.65E-01 | 3.06E-01 | 1.38 | 1.07 | 1.77 |
| hsa-miR-1303 | 0.52 | 0.32 | 0.87 | 4.02E-02 | 7.09E-01 | 6.34E-01 | 1.32E-02 | 4.27E-01 | 3.06E-01 | 1.22 | 1.04 | 1.43 |
| hsa-miR-422a | 0.51 | 0.28 | 0.92 | 3.21E-02 | 8.90E-01 | 6.34E-01 | 1.39E-02 | 3.86E-01 | 3.06E-01 | 1.21 | 1.04 | 1.40 |
| hsa-miR-1260 | 0.69 | 0.50 | 0.97 | 6.69E-02 | 6.83E-01 | 6.34E-01 | 1.62E-02 | 3.93E-01 | 3.06E-01 | 1.45 | 1.07 | 1.97 |
| hsa-miR-1233 | 0.64 | 0.40 | 1.02 | 3.08E-01 | 7.21E-01 | 6.99E-01 | 1.88E-02 | 4.06E-01 | 3.06E-01 | 1.26 | 1.04 | 1.53 |
| hsa-miR-625# | 0.77 | 0.59 | 0.99 | 8.83E-02 | 6.35E-01 | 6.35E-01 | 2.04E-02 | 3.95E-01 | 3.06E-01 | 1.58 | 1.07 | 2.32 |
| rno-miR-7# | 0.60 | 0.37 | 0.97 | 3.68E-02 | 7.14E-01 | 6.34E-01 | 2.10E-02 | 3.69E-01 | 3.06E-01 | 1.22 | 1.03 | 1.44 |
| hsa-miR-1296 | 1.76 | 1.09 | 2.86 | 2.67E-02 | 8.63E-01 | 6.34E-01 | 2.23E-02 | 3.60E-01 | 3.06E-01 | 0.77 | 0.62 | 0.96 |
| hsa-miR-661 | 0.46 | 0.21 | 1.04 | 5.67E-02 | 6.48E-01 | 6.34E-01 | 2.33E-02 | 3.47E-01 | 3.06E-01 | 1.13 | 1.02 | 1.26 |
| hsa-miR-185 | 0.70 | 0.50 | 0.99 | 8.16E-02 | 6.34E-01 | 6.34E-01 | 2.39E-02 | 3.30E-01 | 3.06E-01 | 1.34 | 1.04 | 1.73 |
| hsa-miR-26a-1# | 0.62 | 0.39 | 0.97 | 7.54E-02 | 6.66E-01 | 6.34E-01 | 2.58E-02 | 3.33E-01 | 3.06E-01 | 1.25 | 1.03 | 1.53 |
| hsa-miR-126# | 0.47 | 0.23 | 0.97 | 8.49E-02 | 6.34E-01 | 6.34E-01 | 2.74E-02 | 3.31E-01 | 3.06E-01 | 1.15 | 1.02 | 1.31 |
| hsa-miR-639 | 0.56 | 0.31 | 1.00 | 1.58E-01 | 7.30E-01 | 6.99E-01 | 2.92E-02 | 3.32E-01 | 3.06E-01 | 1.20 | 1.02 | 1.42 |
| hsa-miR-650 | 0.64 | 0.42 | 0.98 | 4.89E-02 | 6.78E-01 | 6.34E-01 | 2.95E-02 | 3.17E-01 | 3.06E-01 | 1.27 | 1.02 | 1.58 |
| hsa-miR-577 | 0.59 | 0.34 | 1.01 | 3.28E-02 | 7.97E-01 | 6.34E-01 | 3.26E-02 | 3.33E-01 | 3.06E-01 | 1.17 | 1.01 | 1.36 |
| has-miR-155 | 0.62 | 0.43 | 0.89 | 1.41E-02 | 5.49E-01 | 5.49E-01 | 3.29E-02 | 3.18E-01 | 3.06E-01 | 1.29 | 1.02 | 1.64 |
| hsa-miR-636 | 0.46 | 0.21 | 1.03 | 9.53E-02 | 6.61E-01 | 6.61E-01 | 3.40E-02 | 3.14E-01 | 3.06E-01 | 1.16 | 1.01 | 1.32 |
| hsa-miR-571 | 0.61 | 0.36 | 1.05 | 1.63E-01 | 7.38E-01 | 6.99E-01 | 3.48E-02 | 3.06E-01 | 3.06E-01 | 1.20 | 1.01 | 1.42 |
| hsa-miR-25 | 0.73 | 0.49 | 1.07 | 2.91E-01 | 7.25E-01 | 6.99E-01 | 3.78E-02 | 3.18E-01 | 3.18E-01 | 1.33 | 1.02 | 1.75 |
| hsa-miR-191 | 0.82 | 0.66 | 1.01 | 6.29E-02 | 6.78E-01 | 6.34E-01 | 5.35E-02 | 4.31E-01 | 3.93E-01 | 1.54 | 0.99 | 2.38 |
| hsa-miR-203 | 0.68 | 0.47 | 0.98 | 5.55E-02 | 6.74E-01 | 6.34E-01 | 5.41E-02 | 4.19E-01 | 3.93E-01 | 1.27 | 1.00 | 1.63 |
| hsa-miR-497 | 1.83 | 1.09 | 3.08 | 1.30E-01 | 7.91E-01 | 6.99E-01 | 5.70E-02 | 4.24E-01 | 3.93E-01 | 0.83 | 0.69 | 1.01 |
| hsa-miR-19a | 0.59 | 0.34 | 1.04 | 1.05E-01 | 6.78E-01 | 6.78E-01 | 5.77E-02 | 4.14E-01 | 3.93E-01 | 1.17 | 0.99 | 1.37 |
| hsa-miR-151-5P | 1.49 | 1.03 | 2.15 | 6.69E-02 | 6.49E-01 | 6.34E-01 | 5.94E-02 | 4.11E-01 | 3.93E-01 | 0.77 | 0.58 | 1.01 |
| hsa-miR-215 | 0.70 | 0.48 | 1.04 | 4.20E-02 | 6.79E-01 | 6.34E-01 | 6.16E-02 | 4.12E-01 | 3.93E-01 | 1.23 | 0.99 | 1.52 |
| hsa-miR-539 | 1.71 | 1.05 | 2.81 | 2.12E-01 | 7.36E-01 | 6.99E-01 | 6.21E-02 | 4.01E-01 | 3.93E-01 | 0.82 | 0.66 | 1.01 |
| hsa-miR-34a | 0.71 | 0.49 | 1.05 | 1.13E-01 | 7.07E-01 | 6.99E-01 | 6.29E-02 | 3.93E-01 | 3.93E-01 | 1.25 | 0.99 | 1.58 |
| hsa-miR-494 | 0.63 | 0.38 | 1.04 | 7.85E-02 | 6.63E-01 | 6.34E-01 | 6.84E-02 | 4.14E-01 | 4.14E-01 | 1.19 | 0.99 | 1.45 |
| hsa-miR-132 | 0.74 | 0.54 | 1.02 | 4.78E-02 | 7.15E-01 | 6.34E-01 | 7.18E-02 | 4.22E-01 | 4.15E-01 | 1.28 | 0.98 | 1.69 |
| hsa-miR-1225-3P | 0.59 | 0.28 | 1.25 | 2.48E-01 | 7.29E-01 | 6.99E-01 | 7.45E-02 | 4.25E-01 | 4.15E-01 | 1.13 | 0.99 | 1.28 |
| hsa-miR-378 | 0.59 | 0.30 | 1.15 | 1.90E-01 | 7.37E-01 | 6.99E-01 | 7.54E-02 | 4.17E-01 | 4.15E-01 | 1.19 | 0.98 | 1.43 |
| hsa-miR-212 | 0.62 | 0.37 | 1.04 | 5.44E-02 | 7.04E-01 | 6.34E-01 | 7.71E-02 | 4.15E-01 | 4.15E-01 | 1.16 | 0.98 | 1.38 |
| hsa-miR-622 | 0.80 | 0.60 | 1.07 | 2.19E-01 | 7.46E-01 | 6.99E-01 | 8.06E-02 | 4.22E-01 | 4.22E-01 | 1.36 | 0.96 | 1.91 |
| hsa-miR-625 | 0.66 | 0.38 | 1.16 | 3.30E-01 | 7.13E-01 | 6.99E-01 | 8.75E-02 | 4.46E-01 | 4.46E-01 | 1.15 | 0.98 | 1.34 |
| hsa-miR-374b | 0.74 | 0.49 | 1.11 | 1.72E-01 | 7.10E-01 | 6.99E-01 | 9.60E-02 | 4.77E-01 | 4.77E-01 | 1.22 | 0.97 | 1.54 |
| hsa-miR-1290 | 0.75 | 0.50 | 1.13 | 3.30E-01 | 7.05E-01 | 6.99E-01 | 1.06E-01 | 5.15E-01 | 5.15E-01 | 1.21 | 0.96 | 1.54 |
| hsa-miR-744 | 0.79 | 0.58 | 1.08 | 7.10E-02 | 6.57E-01 | 6.34E-01 | 1.23E-01 | 5.81E-01 | 5.58E-01 | 1.26 | 0.94 | 1.69 |
| hsa-miR-320B | 0.74 | 0.41 | 1.32 | 2.29E-01 | 7.19E-01 | 6.99E-01 | 1.30E-01 | 5.98E-01 | 5.58E-01 | 1.13 | 0.96 | 1.33 |
| hsa-miR-140-5p | 0.79 | 0.57 | 1.08 | 2.63E-01 | 7.51E-01 | 6.99E-01 | 1.31E-01 | 5.88E-01 | 5.58E-01 | 1.26 | 0.93 | 1.70 |
| hsa-miR-1183 | 0.74 | 0.45 | 1.21 | 5.68E-01 | 7.99E-01 | 7.82E-01 | 1.33E-01 | 5.86E-01 | 5.58E-01 | 1.17 | 0.95 | 1.43 |
| hsa-miR-20b | 0.68 | 0.40 | 1.16 | 1.55E-01 | 7.54E-01 | 6.99E-01 | 1.36E-01 | 5.86E-01 | 5.58E-01 | 1.13 | 0.96 | 1.32 |
| hsa-miR-425 | 0.79 | 0.58 | 1.09 | 3.78E-01 | 7.34E-01 | 7.19E-01 | 1.38E-01 | 5.82E-01 | 5.58E-01 | 1.25 | 0.93 | 1.68 |
| hsa-miR-106b | 0.77 | 0.50 | 1.17 | 2.09E-01 | 7.65E-01 | 6.99E-01 | 1.40E-01 | 5.78E-01 | 5.58E-01 | 1.17 | 0.95 | 1.43 |
| hsa-miR-18a# | 0.67 | 0.41 | 1.10 | 2.44E-01 | 7.29E-01 | 6.99E-01 | 1.42E-01 | 5.73E-01 | 5.58E-01 | 1.14 | 0.96 | 1.36 |
| hsa-miR-638 | 0.71 | 0.41 | 1.21 | 1.58E-01 | 7.48E-01 | 6.99E-01 | 1.44E-01 | 5.69E-01 | 5.58E-01 | 1.13 | 0.96 | 1.33 |
| hsa-miR-374a | 0.73 | 0.43 | 1.22 | 3.00E-01 | 7.36E-01 | 6.99E-01 | 1.47E-01 | 5.71E-01 | 5.58E-01 | 1.15 | 0.95 | 1.38 |
| hsa-miR-29c | 0.56 | 0.23 | 1.34 | 2.55E-01 | 7.40E-01 | 6.99E-01 | 1.49E-01 | 5.64E-01 | 5.58E-01 | 1.08 | 0.97 | 1.21 |
| hsa-miR-410 | 0.75 | 0.50 | 1.12 | 3.00E-01 | 7.27E-01 | 6.99E-01 | 1.50E-01 | 5.58E-01 | 5.58E-01 | 1.18 | 0.94 | 1.49 |
| hsa-miR-552 | 1.78 | 0.93 | 3.39 | 1.69E-01 | 7.46E-01 | 6.99E-01 | 1.59E-01 | 5.80E-01 | 5.72E-01 | 0.89 | 0.76 | 1.05 |
| hsa-miR-140-3p | 0.77 | 0.55 | 1.08 | 1.30E-01 | 7.45E-01 | 6.99E-01 | 1.62E-01 | 5.83E-01 | 5.72E-01 | 1.21 | 0.93 | 1.58 |
| hsa-miR-590-5p | 0.73 | 0.44 | 1.21 | 3.30E-01 | 7.21E-01 | 6.99E-01 | 1.64E-01 | 5.78E-01 | 5.72E-01 | 1.15 | 0.95 | 1.39 |
| hsa-miR-124 | 0.58 | 0.29 | 1.17 | 1.47E-01 | 7.95E-01 | 6.99E-01 | 1.65E-01 | 5.72E-01 | 5.72E-01 | 1.10 | 0.96 | 1.25 |
| hsa-miR-18a | 0.68 | 0.39 | 1.20 | 2.71E-01 | 7.41E-01 | 6.99E-01 | 1.71E-01 | 5.81E-01 | 5.81E-01 | 1.12 | 0.95 | 1.31 |
| hsa-miR-142-3p | 0.73 | 0.47 | 1.15 | 2.12E-01 | 7.49E-01 | 6.99E-01 | 1.75E-01 | 5.84E-01 | 5.81E-01 | 1.15 | 0.94 | 1.41 |
| hsa-miR-500 | 0.75 | 0.47 | 1.19 | 1.78E-01 | 7.19E-01 | 6.99E-01 | 1.77E-01 | 5.81E-01 | 5.81E-01 | 1.14 | 0.94 | 1.37 |
| hsa-miR-328 | 0.64 | 0.32 | 1.28 | 1.05E-01 | 7.01E-01 | 6.78E-01 | 1.82E-01 | 5.88E-01 | 5.88E-01 | 1.09 | 0.96 | 1.25 |
| hsa-miR-1275 | 0.72 | 0.40 | 1.30 | 1.47E-01 | 7.53E-01 | 6.99E-01 | 1.89E-01 | 6.01E-01 | 6.01E-01 | 1.10 | 0.95 | 1.27 |
| hsa-miR-657 | 0.60 | 0.27 | 1.32 | 2.19E-01 | 7.33E-01 | 6.99E-01 | 1.93E-01 | 6.04E-01 | 6.04E-01 | 1.10 | 0.95 | 1.27 |
| hsa-miR-200a# | 0.82 | 0.58 | 1.15 | 4.73E-01 | 7.53E-01 | 7.32E-01 | 2.07E-01 | 6.36E-01 | 6.36E-01 | 1.20 | 0.90 | 1.59 |
| hsa-miR-183# | 1.40 | 0.97 | 2.03 | 1.55E-01 | 7.73E-01 | 6.99E-01 | 2.10E-01 | 6.36E-01 | 6.36E-01 | 0.84 | 0.64 | 1.10 |
| hsa-miR-28-3p | 0.85 | 0.65 | 1.11 | 2.83E-01 | 7.13E-01 | 6.99E-01 | 2.21E-01 | 6.60E-01 | 6.60E-01 | 1.23 | 0.88 | 1.70 |
| hsa-miR-135b | 1.42 | 0.85 | 2.37 | 2.02E-01 | 7.56E-01 | 6.99E-01 | 2.28E-01 | 6.70E-01 | 6.69E-01 | 0.89 | 0.73 | 1.08 |
| hsa-miR-888 | 0.54 | 0.19 | 1.57 | 4.79E-01 | 7.50E-01 | 7.32E-01 | 2.34E-01 | 6.77E-01 | 6.69E-01 | 1.05 | 0.97 | 1.14 |
| hsa-miR-21 | 0.80 | 0.54 | 1.17 | 1.84E-01 | 7.28E-01 | 6.99E-01 | 2.36E-01 | 6.72E-01 | 6.69E-01 | 1.14 | 0.92 | 1.42 |
| hsa-miR-486-5p | 0.79 | 0.52 | 1.19 | 1.45E-01 | 8.04E-01 | 6.99E-01 | 2.41E-01 | 6.76E-01 | 6.69E-01 | 1.16 | 0.91 | 1.49 |
| hsa-miR-886-3p | 0.76 | 0.50 | 1.15 | 1.99E-01 | 7.58E-01 | 6.99E-01 | 2.42E-01 | 6.69E-01 | 6.69E-01 | 1.14 | 0.91 | 1.42 |
| hsa-miR-130a | 0.79 | 0.52 | 1.21 | 3.54E-01 | 7.23E-01 | 7.10E-01 | 2.46E-01 | 6.72E-01 | 6.70E-01 | 1.14 | 0.91 | 1.43 |
| hsa-miR-320 | 0.87 | 0.68 | 1.11 | 2.63E-01 | 7.40E-01 | 6.99E-01 | 2.49E-01 | 6.70E-01 | 6.70E-01 | 1.24 | 0.86 | 1.79 |
| hsa-miR-192 | 0.75 | 0.47 | 1.19 | 2.40E-01 | 7.29E-01 | 6.99E-01 | 2.55E-01 | 6.76E-01 | 6.76E-01 | 1.12 | 0.92 | 1.36 |
| hsa-miR-431 | 0.61 | 0.29 | 1.31 | 4.51E-01 | 7.61E-01 | 7.32E-01 | 2.59E-01 | 6.78E-01 | 6.78E-01 | 1.07 | 0.95 | 1.22 |
| hsa-miR-345 | 0.87 | 0.65 | 1.18 | 3.26E-01 | 7.27E-01 | 6.99E-01 | 2.65E-01 | 6.84E-01 | 6.84E-01 | 1.20 | 0.87 | 1.64 |
| hsa-miR-93 | 0.85 | 0.56 | 1.27 | 3.78E-01 | 7.26E-01 | 7.19E-01 | 2.74E-01 | 6.98E-01 | 6.98E-01 | 1.13 | 0.91 | 1.40 |
| hsa-miR-362-5p | 0.81 | 0.49 | 1.34 | 2.71E-01 | 7.30E-01 | 6.99E-01 | 2.87E-01 | 7.23E-01 | 7.23E-01 | 1.10 | 0.93 | 1.30 |
| hsa-miR-592 | 1.47 | 0.70 | 3.11 | 2.83E-01 | 7.23E-01 | 6.99E-01 | 2.97E-01 | 7.36E-01 | 7.33E-01 | 0.93 | 0.80 | 1.07 |
| hsa-miR-150 | 0.78 | 0.54 | 1.13 | 8.16E-02 | 6.61E-01 | 6.34E-01 | 3.01E-01 | 7.38E-01 | 7.33E-01 | 1.14 | 0.89 | 1.45 |
| hsa-miR-1253 | 0.77 | 0.42 | 1.42 | 5.08E-01 | 7.47E-01 | 7.47E-01 | 3.09E-01 | 7.48E-01 | 7.33E-01 | 1.08 | 0.93 | 1.27 |
| hsa-miR-34a# | 0.84 | 0.58 | 1.22 | 3.35E-01 | 6.99E-01 | 6.99E-01 | 3.10E-01 | 7.41E-01 | 7.33E-01 | 1.14 | 0.89 | 1.46 |
| hsa-miR-186 | 0.87 | 0.65 | 1.15 | 2.37E-01 | 7.29E-01 | 6.99E-01 | 3.11E-01 | 7.35E-01 | 7.33E-01 | 1.18 | 0.85 | 1.64 |
| hsa-miR-487a | 0.67 | 0.34 | 1.31 | 1.47E-01 | 7.74E-01 | 6.99E-01 | 3.17E-01 | 7.39E-01 | 7.33E-01 | 1.07 | 0.94 | 1.22 |
| hsa-miR-99b | 0.85 | 0.60 | 1.21 | 2.26E-01 | 7.19E-01 | 6.99E-01 | 3.22E-01 | 7.42E-01 | 7.33E-01 | 1.16 | 0.87 | 1.55 |
| hsa-miR-195 | 0.82 | 0.56 | 1.20 | 1.69E-01 | 7.29E-01 | 6.99E-01 | 3.25E-01 | 7.40E-01 | 7.33E-01 | 1.12 | 0.90 | 1.40 |
| rno-miR-29c# | 0.76 | 0.48 | 1.22 | 6.58E-01 | 8.03E-01 | 7.82E-01 | 3.28E-01 | 7.38E-01 | 7.33E-01 | 1.11 | 0.90 | 1.35 |
| hsa-miR-192# | 0.80 | 0.52 | 1.22 | 2.79E-01 | 7.22E-01 | 6.99E-01 | 3.29E-01 | 7.33E-01 | 7.33E-01 | 1.11 | 0.90 | 1.37 |
| hsa-miR-15a | 0.78 | 0.46 | 1.32 | 2.26E-01 | 7.31E-01 | 6.99E-01 | 3.38E-01 | 7.44E-01 | 7.38E-01 | 1.09 | 0.92 | 1.29 |
| hsa-miR-1291 | 1.18 | 0.85 | 1.63 | 3.58E-01 | 7.10E-01 | 7.10E-01 | 3.40E-01 | 7.39E-01 | 7.38E-01 | 0.85 | 0.61 | 1.19 |
| hsa-miR-1201 | 1.27 | 0.86 | 1.88 | 3.04E-01 | 7.28E-01 | 6.99E-01 | 3.45E-01 | 7.43E-01 | 7.38E-01 | 0.89 | 0.71 | 1.13 |
| hsa-miR-134 | 1.27 | 0.84 | 1.92 | 7.80E-01 | 8.33E-01 | 8.16E-01 | 3.51E-01 | 7.48E-01 | 7.38E-01 | 0.89 | 0.70 | 1.13 |
| hsa-miR-125b | 0.80 | 0.51 | 1.24 | 1.30E-01 | 7.67E-01 | 6.99E-01 | 3.52E-01 | 7.42E-01 | 7.38E-01 | 1.10 | 0.90 | 1.34 |
| hsa-miR-452 | 0.85 | 0.57 | 1.26 | 4.40E-01 | 7.63E-01 | 7.32E-01 | 3.59E-01 | 7.48E-01 | 7.38E-01 | 1.12 | 0.88 | 1.41 |
| hsa-miR-484 | 0.72 | 0.37 | 1.42 | 1.69E-01 | 7.13E-01 | 6.99E-01 | 3.66E-01 | 7.55E-01 | 7.38E-01 | 1.07 | 0.93 | 1.22 |
| hsa-miR-133a | 0.72 | 0.37 | 1.41 | 7.87E-01 | 8.22E-01 | 8.16E-01 | 3.69E-01 | 7.53E-01 | 7.38E-01 | 1.07 | 0.92 | 1.24 |
| hsa-miR-223 | 0.80 | 0.54 | 1.19 | 2.79E-01 | 7.32E-01 | 6.99E-01 | 3.70E-01 | 7.46E-01 | 7.38E-01 | 1.11 | 0.88 | 1.41 |
| hsa-miR-23a | 0.74 | 0.37 | 1.51 | 2.12E-01 | 7.63E-01 | 6.99E-01 | 3.71E-01 | 7.40E-01 | 7.38E-01 | 1.06 | 0.93 | 1.22 |
| hsa-miR-146a | 0.85 | 0.63 | 1.15 | 3.04E-01 | 7.20E-01 | 6.99E-01 | 3.79E-01 | 7.48E-01 | 7.38E-01 | 1.14 | 0.85 | 1.52 |
| hsa-miR-340 | 0.89 | 0.55 | 1.44 | 8.16E-01 | 8.25E-01 | 8.17E-01 | 3.83E-01 | 7.50E-01 | 7.38E-01 | 1.10 | 0.89 | 1.36 |
| hsa-miR-523 | 0.74 | 0.33 | 1.65 | 5.93E-01 | 8.00E-01 | 7.82E-01 | 3.85E-01 | 7.45E-01 | 7.38E-01 | 1.05 | 0.94 | 1.18 |
| hsa-miR-103 | 0.83 | 0.55 | 1.26 | 3.13E-01 | 7.14E-01 | 6.99E-01 | 3.85E-01 | 7.38E-01 | 7.38E-01 | 1.10 | 0.88 | 1.37 |
| hsa-miR-30a-5p | 1.15 | 0.83 | 1.59 | 5.74E-01 | 7.96E-01 | 7.82E-01 | 3.95E-01 | 7.49E-01 | 7.43E-01 | 0.87 | 0.64 | 1.19 |
| hsa-miR-200a | 0.84 | 0.56 | 1.26 | 3.98E-01 | 7.36E-01 | 7.32E-01 | 3.98E-01 | 7.48E-01 | 7.43E-01 | 1.10 | 0.88 | 1.38 |
| hsa-miR-660 | 0.87 | 0.58 | 1.30 | 6.78E-01 | 7.93E-01 | 7.89E-01 | 4.09E-01 | 7.62E-01 | 7.43E-01 | 1.10 | 0.87 | 1.39 |
| hsa-miR-423-5p | 0.78 | 0.45 | 1.36 | 4.35E-01 | 7.60E-01 | 7.32E-01 | 4.15E-01 | 7.66E-01 | 7.43E-01 | 1.07 | 0.91 | 1.27 |
| hsa-miR-99a | 0.82 | 0.52 | 1.29 | 3.40E-01 | 7.01E-01 | 7.01E-01 | 4.19E-01 | 7.65E-01 | 7.43E-01 | 1.09 | 0.89 | 1.34 |
| hsa-miR-483-3p | 0.88 | 0.42 | 1.85 | 3.58E-01 | 7.17E-01 | 7.10E-01 | 4.21E-01 | 7.61E-01 | 7.43E-01 | 1.06 | 0.92 | 1.21 |
| hsa-miR-532-5p | 0.92 | 0.59 | 1.44 | 9.16E-01 | 8.32E-01 | 8.32E-01 | 4.33E-01 | 7.76E-01 | 7.43E-01 | 1.09 | 0.88 | 1.36 |
| hsa-miR-643 | 0.83 | 0.43 | 1.61 | 3.58E-01 | 7.25E-01 | 7.10E-01 | 4.42E-01 | 7.86E-01 | 7.43E-01 | 1.06 | 0.92 | 1.22 |
| hsa-miR-130b | 0.92 | 0.59 | 1.44 | 3.12E-01 | 7.23E-01 | 6.99E-01 | 4.44E-01 | 7.81E-01 | 7.43E-01 | 1.08 | 0.89 | 1.31 |
| hsa-miR-1247 | 0.79 | 0.34 | 1.82 | 4.29E-01 | 7.58E-01 | 7.32E-01 | 4.45E-01 | 7.77E-01 | 7.43E-01 | 1.05 | 0.92 | 1.20 |
| hsa-miR-331-3p | 0.89 | 0.64 | 1.25 | 3.30E-01 | 7.29E-01 | 6.99E-01 | 4.47E-01 | 7.72E-01 | 7.43E-01 | 1.11 | 0.85 | 1.45 |
| hsa-miR-15b | 0.83 | 0.50 | 1.39 | 2.67E-01 | 7.40E-01 | 6.99E-01 | 4.50E-01 | 7.71E-01 | 7.43E-01 | 1.07 | 0.90 | 1.28 |
| hsa-miR-766 | 0.85 | 0.52 | 1.39 | 3.78E-01 | 7.19E-01 | 7.19E-01 | 4.54E-01 | 7.71E-01 | 7.43E-01 | 1.07 | 0.89 | 1.30 |
| hsa-miR-136# | 1.26 | 0.78 | 2.03 | 6.64E-01 | 7.87E-01 | 7.82E-01 | 4.58E-01 | 7.71E-01 | 7.43E-01 | 0.92 | 0.75 | 1.14 |
| hsa-miR-518f | 0.79 | 0.31 | 2.03 | 5.08E-01 | 7.52E-01 | 7.47E-01 | 4.66E-01 | 7.79E-01 | 7.43E-01 | 1.04 | 0.93 | 1.16 |
| hsa-miR-489 | 0.81 | 0.45 | 1.46 | 4.79E-01 | 7.56E-01 | 7.32E-01 | 4.76E-01 | 7.88E-01 | 7.43E-01 | 1.06 | 0.90 | 1.25 |
| hsa-miR-194 | 0.86 | 0.55 | 1.34 | 4.67E-01 | 7.57E-01 | 7.32E-01 | 4.77E-01 | 7.84E-01 | 7.43E-01 | 1.08 | 0.88 | 1.33 |
| hsa-miR-455-5p | 0.88 | 0.60 | 1.30 | 6.31E-01 | 7.96E-01 | 7.82E-01 | 4.80E-01 | 7.81E-01 | 7.43E-01 | 1.09 | 0.85 | 1.40 |
| hsa-let-7f | 0.89 | 0.53 | 1.52 | 8.87E-01 | 8.29E-01 | 8.22E-01 | 4.80E-01 | 7.75E-01 | 7.43E-01 | 1.07 | 0.89 | 1.28 |
| hsa-miR-28-5p | 0.88 | 0.53 | 1.46 | 7.04E-01 | 8.00E-01 | 7.95E-01 | 4.81E-01 | 7.70E-01 | 7.43E-01 | 1.08 | 0.87 | 1.34 |
| hsa-miR-125a-5p | 0.87 | 0.64 | 1.18 | 6.58E-01 | 7.98E-01 | 7.82E-01 | 4.89E-01 | 7.77E-01 | 7.43E-01 | 1.12 | 0.82 | 1.52 |
| hsa-miR-138 | 0.83 | 0.49 | 1.42 | 7.18E-01 | 8.06E-01 | 8.02E-01 | 4.91E-01 | 7.72E-01 | 7.43E-01 | 1.07 | 0.88 | 1.32 |
| hsa-let-7c | 1.13 | 0.70 | 1.85 | 9.02E-01 | 8.26E-01 | 8.22E-01 | 4.94E-01 | 7.71E-01 | 7.43E-01 | 0.93 | 0.77 | 1.14 |
| hsa-miR-584 | 0.76 | 0.35 | 1.66 | 4.45E-01 | 7.59E-01 | 7.32E-01 | 4.94E-01 | 7.66E-01 | 7.43E-01 | 1.04 | 0.92 | 1.18 |
| hsa-miR-491-5p | 0.92 | 0.68 | 1.24 | 7.46E-01 | 8.09E-01 | 8.09E-01 | 5.04E-01 | 7.74E-01 | 7.43E-01 | 1.12 | 0.81 | 1.55 |
| hsa-miR-222 | 0.89 | 0.66 | 1.20 | 4.56E-01 | 7.51E-01 | 7.32E-01 | 5.04E-01 | 7.68E-01 | 7.43E-01 | 1.11 | 0.81 | 1.53 |
| hsa-miR-127-3p | 0.88 | 0.59 | 1.30 | 5.87E-01 | 7.97E-01 | 7.82E-01 | 5.04E-01 | 7.62E-01 | 7.43E-01 | 1.09 | 0.85 | 1.40 |
| hsa-miR-518b | 0.81 | 0.41 | 1.59 | 4.24E-01 | 7.69E-01 | 7.32E-01 | 5.06E-01 | 7.60E-01 | 7.43E-01 | 1.05 | 0.91 | 1.21 |
| hsa-miR-146b-5p | 0.87 | 0.66 | 1.15 | 2.22E-01 | 7.32E-01 | 6.99E-01 | 5.11E-01 | 7.62E-01 | 7.43E-01 | 1.11 | 0.81 | 1.54 |
| hsa-miR-769-5p | 1.10 | 0.79 | 1.53 | 5.43E-01 | 7.70E-01 | 7.70E-01 | 5.13E-01 | 7.59E-01 | 7.43E-01 | 0.90 | 0.67 | 1.22 |
| hsa-miR-30a-3p | 0.90 | 0.67 | 1.23 | 5.19E-01 | 7.53E-01 | 7.53E-01 | 5.14E-01 | 7.54E-01 | 7.43E-01 | 1.11 | 0.82 | 1.49 |
| hsa-miR-143 | 0.86 | 0.57 | 1.30 | 6.06E-01 | 8.11E-01 | 7.82E-01 | 5.15E-01 | 7.50E-01 | 7.43E-01 | 1.08 | 0.86 | 1.35 |
| hsa-miR-17 | 0.89 | 0.60 | 1.31 | 6.25E-01 | 8.04E-01 | 7.82E-01 | 5.16E-01 | 7.46E-01 | 7.43E-01 | 1.08 | 0.85 | 1.38 |
| hsa-miR-411 | 0.85 | 0.53 | 1.36 | 6.06E-01 | 8.06E-01 | 7.82E-01 | 5.24E-01 | 7.52E-01 | 7.43E-01 | 1.06 | 0.88 | 1.29 |
| hsa-miR-19b | 0.85 | 0.52 | 1.37 | 4.24E-01 | 7.77E-01 | 7.32E-01 | 5.27E-01 | 7.51E-01 | 7.43E-01 | 1.07 | 0.87 | 1.30 |
| hsa-let-7b | 0.91 | 0.60 | 1.37 | 3.35E-01 | 7.07E-01 | 6.99E-01 | 5.28E-01 | 7.46E-01 | 7.43E-01 | 1.07 | 0.86 | 1.34 |
| hsa-miR-652 | 0.89 | 0.59 | 1.34 | 3.88E-01 | 7.24E-01 | 7.24E-01 | 5.30E-01 | 7.44E-01 | 7.43E-01 | 1.07 | 0.86 | 1.33 |
| hsa-miR-200b | 0.88 | 0.58 | 1.34 | 6.25E-01 | 7.98E-01 | 7.82E-01 | 5.34E-01 | 7.44E-01 | 7.43E-01 | 1.07 | 0.86 | 1.35 |
| hsa-miR-425# | 1.17 | 0.75 | 1.81 | 8.95E-01 | 8.23E-01 | 8.22E-01 | 5.39E-01 | 7.46E-01 | 7.43E-01 | 0.94 | 0.75 | 1.16 |
| hsa-miR-100 | 0.88 | 0.61 | 1.26 | 6.25E-01 | 8.09E-01 | 7.82E-01 | 5.47E-01 | 7.52E-01 | 7.43E-01 | 1.10 | 0.81 | 1.48 |
| hsa-let-7d | 0.94 | 0.69 | 1.28 | 4.45E-01 | 7.65E-01 | 7.32E-01 | 5.48E-01 | 7.47E-01 | 7.43E-01 | 1.09 | 0.82 | 1.46 |
| hsa-miR-27b# | 1.27 | 0.74 | 2.18 | 3.73E-01 | 7.31E-01 | 7.19E-01 | 5.51E-01 | 7.47E-01 | 7.43E-01 | 0.94 | 0.78 | 1.14 |
| hsa-miR-20a | 0.86 | 0.50 | 1.47 | 4.90E-01 | 7.38E-01 | 7.32E-01 | 5.55E-01 | 7.47E-01 | 7.43E-01 | 1.05 | 0.88 | 1.26 |
| hsa-miR-302a | 1.49 | 0.49 | 4.49 | 9.82E-01 | 8.51E-01 | 8.48E-01 | 5.56E-01 | 7.43E-01 | 7.43E-01 | 0.97 | 0.89 | 1.06 |
| hsa-miR-30d | 1.13 | 0.80 | 1.60 | 7.04E-01 | 7.95E-01 | 7.95E-01 | 5.73E-01 | 7.60E-01 | 7.55E-01 | 0.92 | 0.69 | 1.22 |
| hsa-miR-19b-1# | 0.84 | 0.37 | 1.90 | 4.79E-01 | 7.44E-01 | 7.32E-01 | 5.73E-01 | 7.55E-01 | 7.55E-01 | 1.04 | 0.91 | 1.18 |
| hsa-miR-214# | 1.12 | 0.82 | 1.53 | 7.80E-01 | 8.24E-01 | 8.16E-01 | 5.77E-01 | 7.55E-01 | 7.55E-01 | 0.91 | 0.66 | 1.26 |
| hsa-miR-454 | 0.92 | 0.63 | 1.34 | 5.74E-01 | 8.02E-01 | 7.82E-01 | 5.90E-01 | 7.67E-01 | 7.62E-01 | 1.07 | 0.84 | 1.35 |
| hsa-miR-342 | 0.91 | 0.67 | 1.24 | 4.51E-01 | 7.48E-01 | 7.32E-01 | 5.90E-01 | 7.62E-01 | 7.62E-01 | 1.09 | 0.79 | 1.50 |
| hsa-miR-222# | 0.93 | 0.65 | 1.34 | 6.64E-01 | 7.82E-01 | 7.82E-01 | 6.01E-01 | 7.71E-01 | 7.68E-01 | 1.07 | 0.83 | 1.39 |
| hsa-miR-1227 | 0.88 | 0.54 | 1.45 | 8.02E-01 | 8.19E-01 | 8.17E-01 | 6.03E-01 | 7.68E-01 | 7.68E-01 | 1.05 | 0.87 | 1.28 |
| hsa-miR-429 | 0.89 | 0.55 | 1.43 | 7.39E-01 | 8.11E-01 | 8.09E-01 | 6.06E-01 | 7.68E-01 | 7.68E-01 | 1.05 | 0.86 | 1.28 |
| hsa-miR-382 | 0.91 | 0.49 | 1.71 | 4.29E-01 | 7.65E-01 | 7.32E-01 | 6.15E-01 | 7.73E-01 | 7.73E-01 | 1.04 | 0.90 | 1.20 |
| hsa-miR-193b | 1.03 | 0.80 | 1.32 | 6.64E-01 | 7.96E-01 | 7.82E-01 | 6.22E-01 | 7.77E-01 | 7.74E-01 | 0.91 | 0.62 | 1.33 |
| hsa-miR-31 | 0.84 | 0.45 | 1.57 | 4.67E-01 | 7.50E-01 | 7.32E-01 | 6.23E-01 | 7.74E-01 | 7.74E-01 | 1.04 | 0.89 | 1.21 |
| hsa-miR-628-5p | 1.20 | 0.62 | 2.31 | 6.06E-01 | 8.00E-01 | 7.82E-01 | 6.43E-01 | 7.93E-01 | 7.89E-01 | 0.96 | 0.82 | 1.13 |
| hsa-miR-10a | 0.88 | 0.55 | 1.42 | 9.89E-01 | 8.54E-01 | 8.48E-01 | 6.51E-01 | 7.98E-01 | 7.89E-01 | 1.05 | 0.85 | 1.30 |
| hsa-miR-145# | 1.09 | 0.71 | 1.69 | 8.66E-01 | 8.20E-01 | 8.20E-01 | 6.53E-01 | 7.95E-01 | 7.89E-01 | 0.95 | 0.77 | 1.18 |
| hsa-miR-224 | 1.16 | 0.71 | 1.89 | 3.88E-01 | 7.31E-01 | 7.24E-01 | 6.56E-01 | 7.94E-01 | 7.89E-01 | 0.95 | 0.77 | 1.17 |
| hsa-miR-337-5p | 0.90 | 0.53 | 1.51 | 9.96E-01 | 8.52E-01 | 8.48E-01 | 6.59E-01 | 7.93E-01 | 7.89E-01 | 1.05 | 0.86 | 1.27 |
| hsa-miR-106a | 0.92 | 0.62 | 1.36 | 6.51E-01 | 8.00E-01 | 7.82E-01 | 6.60E-01 | 7.89E-01 | 7.89E-01 | 1.06 | 0.83 | 1.35 |
| hsa-miR-22 | 0.91 | 0.52 | 1.60 | 4.51E-01 | 7.55E-01 | 7.32E-01 | 6.65E-01 | 7.90E-01 | 7.90E-01 | 1.03 | 0.89 | 1.20 |
| hsa-miR-365 | 1.16 | 0.71 | 1.90 | 6.84E-01 | 7.91E-01 | 7.89E-01 | 6.74E-01 | 7.95E-01 | 7.95E-01 | 0.96 | 0.78 | 1.18 |
| hsa-miR-375 | 0.92 | 0.48 | 1.73 | 6.91E-01 | 7.89E-01 | 7.89E-01 | 6.81E-01 | 8.00E-01 | 8.00E-01 | 1.03 | 0.89 | 1.20 |
| hsa-miR-141 | 0.94 | 0.63 | 1.39 | 9.02E-01 | 8.22E-01 | 8.22E-01 | 6.93E-01 | 8.09E-01 | 8.07E-01 | 1.05 | 0.83 | 1.34 |
| hsa-miR-532-3p | 0.97 | 0.66 | 1.43 | 9.89E-01 | 8.50E-01 | 8.48E-01 | 6.96E-01 | 8.07E-01 | 8.07E-01 | 1.05 | 0.82 | 1.34 |
| hsa-miR-10b | 1.13 | 0.69 | 1.84 | 7.59E-01 | 8.15E-01 | 8.15E-01 | 7.05E-01 | 8.12E-01 | 8.07E-01 | 0.96 | 0.78 | 1.18 |
| hsa-miR-26b | 0.93 | 0.56 | 1.54 | 6.84E-01 | 7.96E-01 | 7.89E-01 | 7.08E-01 | 8.12E-01 | 8.07E-01 | 1.04 | 0.86 | 1.25 |
| hsa-miR-605 | 1.15 | 0.64 | 2.06 | 5.43E-01 | 7.76E-01 | 7.70E-01 | 7.10E-01 | 8.09E-01 | 8.07E-01 | 0.97 | 0.82 | 1.14 |
| hsa-miR-181c | 0.95 | 0.55 | 1.65 | 9.74E-01 | 8.60E-01 | 8.48E-01 | 7.12E-01 | 8.07E-01 | 8.07E-01 | 1.03 | 0.87 | 1.23 |
| hsa-miR-214 | 0.94 | 0.70 | 1.25 | 6.12E-01 | 8.03E-01 | 7.82E-01 | 7.31E-01 | 8.23E-01 | 8.11E-01 | 1.06 | 0.76 | 1.47 |
| hsa-miR-95 | 1.11 | 0.68 | 1.81 | 8.16E-01 | 8.17E-01 | 8.17E-01 | 7.34E-01 | 8.21E-01 | 8.11E-01 | 0.97 | 0.79 | 1.18 |
| hsa-miR-34b | 0.97 | 0.62 | 1.51 | 7.32E-01 | 8.08E-01 | 8.08E-01 | 7.55E-01 | 8.41E-01 | 8.11E-01 | 1.03 | 0.84 | 1.28 |
| hsa-miR-1244 | 0.91 | 0.63 | 1.33 | 3.17E-01 | 7.16E-01 | 6.99E-01 | 7.57E-01 | 8.37E-01 | 8.11E-01 | 1.04 | 0.82 | 1.31 |
| hsa-miR-1243 | 0.95 | 0.36 | 2.51 | 2.79E-01 | 7.42E-01 | 6.99E-01 | 7.60E-01 | 8.37E-01 | 8.11E-01 | 1.01 | 0.93 | 1.11 |
| hsa-miR-223# | 1.06 | 0.69 | 1.63 | 5.13E-01 | 7.50E-01 | 7.50E-01 | 7.65E-01 | 8.37E-01 | 8.11E-01 | 0.97 | 0.77 | 1.21 |
| hsa-miR-708 | 0.94 | 0.65 | 1.37 | 6.18E-01 | 8.06E-01 | 7.82E-01 | 7.67E-01 | 8.34E-01 | 8.11E-01 | 1.04 | 0.82 | 1.30 |
| hsa-miR-18b | 0.89 | 0.40 | 1.97 | 4.84E-01 | 7.41E-01 | 7.32E-01 | 7.69E-01 | 8.32E-01 | 8.11E-01 | 1.02 | 0.89 | 1.17 |
| hsa-miR-193a-3p | 1.14 | 0.70 | 1.85 | 8.80E-01 | 8.30E-01 | 8.22E-01 | 7.69E-01 | 8.27E-01 | 8.11E-01 | 0.97 | 0.80 | 1.18 |
| hsa-miR-139-5p | 0.86 | 0.54 | 1.37 | 5.80E-01 | 7.99E-01 | 7.82E-01 | 7.70E-01 | 8.24E-01 | 8.11E-01 | 1.03 | 0.85 | 1.25 |
| hsa-miR-331-5p | 0.95 | 0.34 | 2.69 | 8.59E-01 | 8.30E-01 | 8.20E-01 | 7.72E-01 | 8.21E-01 | 8.11E-01 | 1.02 | 0.91 | 1.13 |
| hsa-miR-886-5p | 0.93 | 0.65 | 1.34 | 6.38E-01 | 7.89E-01 | 7.82E-01 | 7.76E-01 | 8.21E-01 | 8.11E-01 | 1.04 | 0.80 | 1.34 |
| hsa-miR-627 | 0.90 | 0.25 | 3.23 | 8.30E-01 | 8.27E-01 | 8.20E-01 | 7.84E-01 | 8.25E-01 | 8.11E-01 | 1.01 | 0.94 | 1.09 |
| hsa-miR-106b# | 1.01 | 0.65 | 1.56 | 4.62E-01 | 7.54E-01 | 7.32E-01 | 7.86E-01 | 8.23E-01 | 8.11E-01 | 1.03 | 0.84 | 1.26 |
| hsa-miR-370 | 0.89 | 0.54 | 1.49 | 5.80E-01 | 7.94E-01 | 7.82E-01 | 7.87E-01 | 8.19E-01 | 8.11E-01 | 1.03 | 0.86 | 1.23 |
| hsa-miR-21# | 0.95 | 0.73 | 1.24 | 4.84E-01 | 7.35E-01 | 7.32E-01 | 7.87E-01 | 8.15E-01 | 8.11E-01 | 1.05 | 0.73 | 1.51 |
| hsa-miR-361-5p | 1.10 | 0.67 | 1.80 | 6.38E-01 | 7.99E-01 | 7.82E-01 | 7.90E-01 | 8.13E-01 | 8.11E-01 | 0.97 | 0.79 | 1.19 |
| hsa-miR-30b | 1.05 | 0.72 | 1.53 | 6.91E-01 | 7.94E-01 | 7.89E-01 | 7.98E-01 | 8.18E-01 | 8.11E-01 | 0.96 | 0.73 | 1.27 |
| hsa-miR-199a-3p | 1.06 | 0.74 | 1.53 | 9.74E-01 | 8.53E-01 | 8.48E-01 | 8.05E-01 | 8.20E-01 | 8.11E-01 | 0.97 | 0.74 | 1.26 |
| hsa-miR-29a | 0.96 | 0.66 | 1.40 | 8.16E-01 | 8.21E-01 | 8.17E-01 | 8.06E-01 | 8.17E-01 | 8.11E-01 | 1.03 | 0.80 | 1.33 |
| hsa-miR-520c-3p | 1.05 | 0.49 | 2.26 | 8.09E-01 | 8.22E-01 | 8.17E-01 | 8.12E-01 | 8.19E-01 | 8.11E-01 | 1.02 | 0.89 | 1.17 |
| hsa-miR-145 | 1.05 | 0.68 | 1.61 | 7.80E-01 | 8.28E-01 | 8.16E-01 | 8.13E-01 | 8.16E-01 | 8.11E-01 | 0.97 | 0.77 | 1.23 |
| hsa-miR-663B | 0.99 | 0.72 | 1.34 | 5.31E-01 | 7.64E-01 | 7.64E-01 | 8.14E-01 | 8.13E-01 | 8.11E-01 | 1.04 | 0.77 | 1.39 |
| hsa-miR-10b# | 1.05 | 0.74 | 1.50 | 7.87E-01 | 8.18E-01 | 8.16E-01 | 8.17E-01 | 8.11E-01 | 8.11E-01 | 0.97 | 0.74 | 1.27 |
| hsa-miR-16 | 0.92 | 0.66 | 1.29 | 6.31E-01 | 8.01E-01 | 7.82E-01 | 8.31E-01 | 8.21E-01 | 8.18E-01 | 1.03 | 0.78 | 1.36 |
| hsa-miR-27b | 0.97 | 0.73 | 1.30 | 9.31E-01 | 8.41E-01 | 8.40E-01 | 8.32E-01 | 8.18E-01 | 8.18E-01 | 1.04 | 0.73 | 1.49 |
| hsa-miR-1248 | 1.04 | 0.62 | 1.75 | 9.38E-01 | 8.40E-01 | 8.40E-01 | 8.44E-01 | 8.25E-01 | 8.25E-01 | 0.98 | 0.82 | 1.18 |
| hsa-miR-26b# | 0.91 | 0.55 | 1.52 | 4.79E-01 | 7.38E-01 | 7.32E-01 | 8.55E-01 | 8.32E-01 | 8.32E-01 | 1.02 | 0.85 | 1.22 |
| hsa-miR-942 | 1.04 | 0.73 | 1.49 | 8.66E-01 | 8.28E-01 | 8.20E-01 | 8.64E-01 | 8.37E-01 | 8.32E-01 | 0.98 | 0.74 | 1.29 |
| hsa-miR-550 | 1.04 | 0.75 | 1.43 | 7.53E-01 | 8.12E-01 | 8.12E-01 | 8.65E-01 | 8.33E-01 | 8.32E-01 | 0.97 | 0.70 | 1.34 |
| hsa-miR-339-5p | 0.96 | 0.56 | 1.65 | 9.53E-01 | 8.45E-01 | 8.45E-01 | 8.68E-01 | 8.32E-01 | 8.32E-01 | 1.02 | 0.84 | 1.23 |
| hsa-miR-92a | 0.96 | 0.65 | 1.41 | 9.38E-01 | 8.43E-01 | 8.40E-01 | 8.73E-01 | 8.33E-01 | 8.33E-01 | 1.02 | 0.79 | 1.31 |
| hsa-miR-483-5p | 1.13 | 0.39 | 3.28 | 8.51E-01 | 8.31E-01 | 8.20E-01 | 8.78E-01 | 8.34E-01 | 8.33E-01 | 1.01 | 0.92 | 1.11 |
| hsa-miR-15b# | 1.06 | 0.51 | 2.18 | 7.46E-01 | 8.13E-01 | 8.09E-01 | 8.82E-01 | 8.33E-01 | 8.33E-01 | 0.99 | 0.87 | 1.13 |
| hsa-miR-574-3p | 0.96 | 0.74 | 1.24 | 4.90E-01 | 7.32E-01 | 7.32E-01 | 8.89E-01 | 8.35E-01 | 8.35E-01 | 1.03 | 0.71 | 1.48 |
| hsa-miR-339-3p | 0.97 | 0.75 | 1.25 | 7.25E-01 | 8.05E-01 | 8.05E-01 | 8.94E-01 | 8.36E-01 | 8.36E-01 | 1.03 | 0.71 | 1.48 |
| hsa-miR-148b# | 0.99 | 0.59 | 1.66 | 8.51E-01 | 8.35E-01 | 8.20E-01 | 9.01E-01 | 8.39E-01 | 8.39E-01 | 1.01 | 0.83 | 1.23 |
| hsa-miR-376c | 0.97 | 0.67 | 1.41 | 9.96E-01 | 8.49E-01 | 8.48E-01 | 9.11E-01 | 8.44E-01 | 8.44E-01 | 1.01 | 0.78 | 1.31 |
| hsa-miR-30c | 0.99 | 0.69 | 1.42 | 9.53E-01 | 8.49E-01 | 8.45E-01 | 9.32E-01 | 8.59E-01 | 8.46E-01 | 1.01 | 0.77 | 1.33 |
| hsa-miR-148a | 1.02 | 0.69 | 1.52 | 8.95E-01 | 8.27E-01 | 8.22E-01 | 9.32E-01 | 8.55E-01 | 8.46E-01 | 0.99 | 0.76 | 1.29 |
| hsa-miR-29b | 1.09 | 0.64 | 1.85 | 8.37E-01 | 8.29E-01 | 8.20E-01 | 9.36E-01 | 8.55E-01 | 8.46E-01 | 0.99 | 0.83 | 1.19 |
| hsa-let-7a | 0.99 | 0.63 | 1.56 | 6.38E-01 | 7.94E-01 | 7.82E-01 | 9.37E-01 | 8.52E-01 | 8.46E-01 | 1.01 | 0.82 | 1.25 |
| hsa-miR-221 | 1.05 | 0.70 | 1.55 | 8.66E-01 | 8.24E-01 | 8.20E-01 | 9.46E-01 | 8.56E-01 | 8.46E-01 | 1.01 | 0.80 | 1.27 |
| hsa-let-7e | 1.00 | 0.72 | 1.38 | 8.95E-01 | 8.31E-01 | 8.22E-01 | 9.49E-01 | 8.55E-01 | 8.46E-01 | 1.01 | 0.75 | 1.36 |
| hsa-miR-27a# | 0.94 | 0.66 | 1.35 | 4.24E-01 | 7.62E-01 | 7.32E-01 | 9.51E-01 | 8.53E-01 | 8.46E-01 | 1.01 | 0.79 | 1.28 |
| hsa-miR-433 | 1.03 | 0.48 | 2.20 | 6.64E-01 | 7.91E-01 | 7.82E-01 | 9.53E-01 | 8.51E-01 | 8.46E-01 | 1.00 | 0.85 | 1.17 |
| hsa-miR-196b | 1.01 | 0.60 | 1.70 | 9.74E-01 | 8.56E-01 | 8.48E-01 | 9.57E-01 | 8.50E-01 | 8.46E-01 | 1.01 | 0.84 | 1.21 |
| hsa-miR-22# | 0.96 | 0.58 | 1.59 | 9.82E-01 | 8.55E-01 | 8.48E-01 | 9.61E-01 | 8.50E-01 | 8.46E-01 | 1.00 | 0.82 | 1.20 |
| hsa-miR-31# | 1.00 | 0.53 | 1.89 | 8.51E-01 | 8.27E-01 | 8.20E-01 | 9.65E-01 | 8.49E-01 | 8.46E-01 | 1.00 | 0.87 | 1.16 |
| hsa-miR-193a-5p | 1.00 | 0.62 | 1.61 | 7.87E-01 | 8.27E-01 | 8.16E-01 | 9.65E-01 | 8.46E-01 | 8.46E-01 | 1.00 | 0.82 | 1.23 |
| hsa-miR-182 | 1.07 | 0.64 | 1.77 | 6.58E-01 | 7.93E-01 | 7.82E-01 | 9.72E-01 | 8.48E-01 | 8.46E-01 | 1.00 | 0.82 | 1.21 |
| hsa-miR-151-3p | 1.01 | 0.79 | 1.29 | 8.80E-01 | 8.26E-01 | 8.22E-01 | 9.77E-01 | 8.48E-01 | 8.46E-01 | 0.99 | 0.65 | 1.51 |
| hsa-miR-181a | 1.03 | 0.72 | 1.49 | 7.18E-01 | 8.02E-01 | 8.02E-01 | 9.89E-01 | 8.55E-01 | 8.46E-01 | 1.00 | 0.77 | 1.30 |
| hsa-miR-218 | 0.99 | 0.62 | 1.59 | 8.44E-01 | 8.32E-01 | 8.20E-01 | 9.91E-01 | 8.53E-01 | 8.46E-01 | 1.00 | 0.82 | 1.22 |
| hsa-miR-505# | 1.02 | 0.71 | 1.46 | 8.66E-01 | 8.32E-01 | 8.20E-01 | 9.97E-01 | 8.54E-01 | 8.46E-01 | 1.00 | 0.77 | 1.30 |
| hsa-miR-451 | 1.02 | 0.68 | 1.53 | 1.00E+00 | 8.48E-01 | 8.48E-01 | 9.97E-01 | 8.51E-01 | 8.46E-01 | 1.00 | 0.80 | 1.25 |
| hsa-miR-409-3p | 0.99 | 0.74 | 1.34 | 7.94E-01 | 8.16E-01 | 8.16E-01 | 9.99E-01 | 8.49E-01 | 8.46E-01 | 1.00 | 0.72 | 1.38 |
| hsa-miR-376a | 1.02 | 0.68 | 1.53 | 7.94E-01 | 8.21E-01 | 8.16E-01 | 1.00E+00 | 8.46E-01 | 8.46E-01 | 1.00 | 0.80 | 1.25 |

Table 3. MiRNA expression according to histological grade and pT stage.

| **miRNA name** | **Grade=1 dCt** | | **Grade >=2 dCt** | | **Fold  change** | **FC  low CI** | **FC  high CI** | **Modified T-test** | |
| --- | --- | --- | --- | --- | --- | --- | --- | --- | --- |
|  | **Mean** | **STD** | **Mean** | **STD** |  |  |  | **P-value** | **FDR** |
| hsa-miR-138 | 6.81 | 0.88 | 8.02 | 1.77 | 0.43 | 0.27 | 0.69 | 0.003 | 0.58 |
| hsa-miR-134 | 9.11 | 1.03 | 10.13 | 1.43 | 0.49 | 0.30 | 0.80 | 0.012 | 0.67 |
| hsa-miR-34a# | 5.30 | 1.01 | 6.27 | 1.18 | 0.51 | 0.32 | 0.81 | 0.013 | 0.67 |
| hsa-miR-378 | 0.59 | 3.08 | 2.73 | 1.92 | 0.23 | 0.06 | 0.85 | 0.018 | 0.67 |
| hsa-miR-376a | 8.32 | 0.95 | 9.20 | 1.39 | 0.54 | 0.34 | 0.86 | 0.023 | 0.67 |
| hsa-miR-361-5p | 7.61 | 0.86 | 8.44 | 1.65 | 0.56 | 0.36 | 0.88 | 0.027 | 0.67 |
| hsa-miR-126# | 5.05 | 1.36 | 6.20 | 2.35 | 0.45 | 0.23 | 0.89 | 0.028 | 0.67 |
| hsa-miR-196b | 1.67 | 1.70 | 2.85 | 1.60 | 0.44 | 0.21 | 0.93 | 0.032 | 0.67 |
| hsa-miR-1243 | 3.05 | 5.52 | -0.16 | 2.62 | 9.23 | 0.89 | 95.26 | 0.034 | 0.67 |
| hsa-miR-124 | 9.15 | 1.65 | 7.88 | 2.43 | 2.40 | 1.08 | 5.31 | 0.034 | 0.67 |
| hsa-miR-215 | 2.92 | 0.68 | 3.59 | 1.30 | 0.63 | 0.44 | 0.90 | 0.037 | 0.67 |
| hsa-miR-186 | 2.26 | 0.84 | 2.94 | 0.91 | 0.62 | 0.42 | 0.91 | 0.040 | 0.67 |
| hsa-miR-376c | 5.38 | 1.07 | 6.18 | 1.18 | 0.57 | 0.35 | 0.93 | 0.040 | 0.67 |
| hsa-miR-146b-5p | 0.85 | 0.79 | 1.50 | 0.91 | 0.64 | 0.44 | 0.92 | 0.045 | 0.69 |
|  |  |  |  |  |  |  |  |  |  |
| **miRNA name** | **pT=2 dCt** | | **pT=3 dCt** | | **Fold  change** | **FC  low CI** | **FC  high CI** | **Modified T-test** | |
|  | **Mean** | **STD** | **Mean** | **STD** |  |  |  | **P-value** | **FDR** |
| hsa-miR-215 | 3.08 | 0.95 | 3.96 | 1.45 | 0.55 | 0.38 | 0.79 | 0.002 | 0.25 |
| hsa-miR-639 | 8.85 | 1.58 | 9.96 | 2.05 | 0.46 | 0.26 | 0.80 | 0.006 | 0.25 |
| hsa-miR-483-3p | 6.46 | 2.17 | 7.82 | 2.41 | 0.39 | 0.19 | 0.78 | 0.007 | 0.25 |
| hsa-miR-345 | 4.58 | 0.71 | 5.12 | 1.10 | 0.69 | 0.52 | 0.91 | 0.012 | 0.25 |
| hsa-miR-124 | 7.42 | 2.48 | 8.69 | 2.12 | 0.42 | 0.21 | 0.84 | 0.013 | 0.25 |
| hsa-miR-145# | 7.44 | 1.30 | 8.20 | 1.50 | 0.59 | 0.38 | 0.90 | 0.015 | 0.25 |
| hsa-miR-192 | 0.82 | 1.15 | 1.62 | 1.73 | 0.58 | 0.37 | 0.90 | 0.015 | 0.25 |
| hsa-miR-577 | 8.22 | 1.69 | 9.17 | 1.84 | 0.52 | 0.31 | 0.89 | 0.016 | 0.25 |
| hsa-miR-451 | 7.73 | 1.35 | 8.43 | 1.32 | 0.62 | 0.41 | 0.92 | 0.020 | 0.25 |
| hsa-miR-494 | 2.17 | 1.79 | 2.99 | 1.46 | 0.57 | 0.35 | 0.93 | 0.024 | 0.25 |
| hsa-miR-362-5p | 7.03 | 1.33 | 7.83 | 1.83 | 0.57 | 0.35 | 0.93 | 0.024 | 0.25 |
| hsa-miR-200a# | 6.91 | 1.02 | 7.46 | 1.12 | 0.68 | 0.49 | 0.95 | 0.026 | 0.25 |
| hsa-miR-1244 | 6.76 | 1.31 | 7.38 | 1.17 | 0.65 | 0.45 | 0.95 | 0.028 | 0.25 |
| hsa-miR-18a | 7.60 | 1.46 | 8.45 | 2.02 | 0.55 | 0.33 | 0.94 | 0.028 | 0.25 |
| hsa-miR-375 | 6.31 | 1.84 | 7.27 | 2.18 | 0.51 | 0.28 | 0.94 | 0.028 | 0.25 |
| hsa-miR-210 | 3.36 | 1.00 | 3.94 | 1.33 | 0.67 | 0.47 | 0.95 | 0.029 | 0.25 |
| hsa-miR-320 | 1.37 | 0.68 | 1.77 | 0.86 | 0.75 | 0.60 | 0.95 | 0.029 | 0.25 |
| hsa-miR-584 | 6.47 | 2.32 | 7.61 | 2.62 | 0.45 | 0.21 | 0.96 | 0.034 | 0.27 |
| hsa-miR-1227 | 9.97 | 1.28 | 10.69 | 1.81 | 0.61 | 0.38 | 0.97 | 0.037 | 0.28 |
| hsa-miR-20b | 6.98 | 1.42 | 7.76 | 1.96 | 0.58 | 0.35 | 0.98 | 0.039 | 0.28 |
| hsa-miR-625# | 6.29 | 0.74 | 6.69 | 0.90 | 0.76 | 0.59 | 0.97 | 0.041 | 0.29 |
