# Supplementary material for: Prognostic value of 5-microRNA based signature in T2-T3N0 colon cancer
Source: Clin Exp Metastasis. 2016 Aug 2;33(8):765–73. doi: 10.1007/s10585-016-9810-1 (PMC5110606; doi:10.1007/s10585-016-9810-1)
Supplement: Supplementary file 2 — Supplementary material 2 (DOCX 192 kb) [file 10585_2016_9810_MOESM2_ESM.docx]

**Supplementary material**

**The choice of miRNA expression positivity threshold**

For each miRNA the percentage of samples with no amplification signal (Ct >40) and the median value of raw Ct were computed. The relationship between these two statistics is shown on the graph below. MiRNA with lower expression are more likely to produce no amplification signal. In this study, only miRNAs with no amplification signal in less than 5% of the samples were included in the analysis, and thus, the low expression miRNAs that might have included those with unreliable measurements were removed. Such operation gives similar results to that whereby the Ct threshold is set to 35. In theory, the approach used in this study allows for analysis the miRNAs with truly positive low expression in the majority of samples rather than those with false positive expression signal. However, in fact, no such miRNAs were detected.


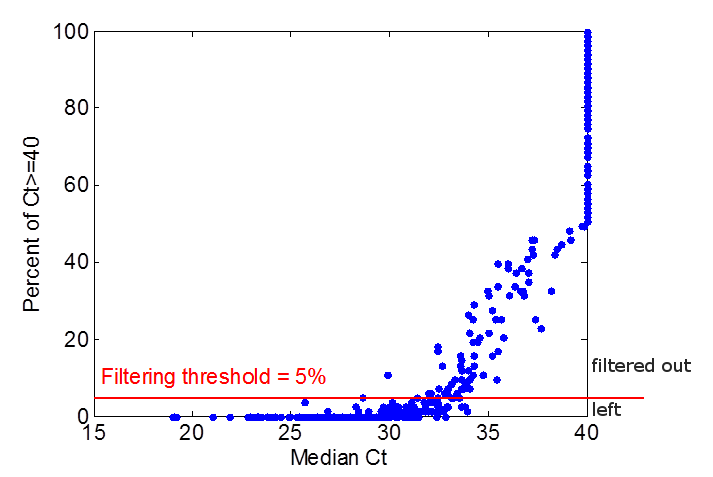


In the table below the distribution of Ct values for miRNAs that were included in the prognostic signature (miR-135b, miR-185, miR-539, miR-1296 and miR-572), miRNAs found prognostic in Cox multivariate analysis (miR-1300 and miR-939) and miR-592 that was associated with MMR status are presented. The upper quartile (splits off the highest 25% of data from the lowest 75%) is lower than 35 for all miRNAs. As can be seen, setting the threshold value to Ct=35 (red line) does not affect significantly the distribution of Ct values.

| miRNA | Distribution of raw Ct values | Comparison of raw Ct values between non-relapse (0) and relapse groups (1) |
| --- | --- | --- |
| miR-135b | 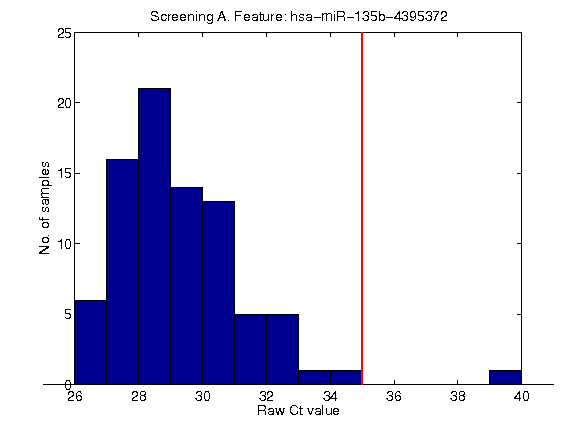 | 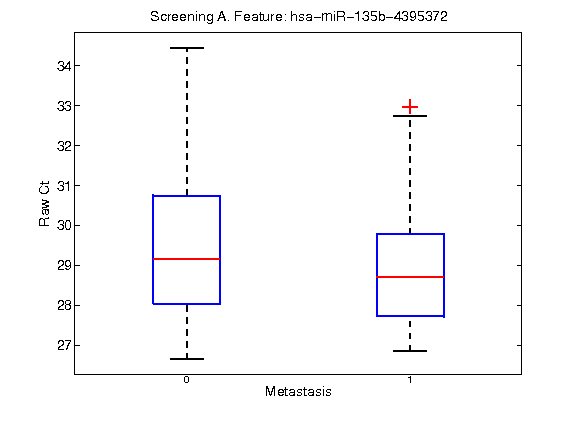 |
| miR-185 | 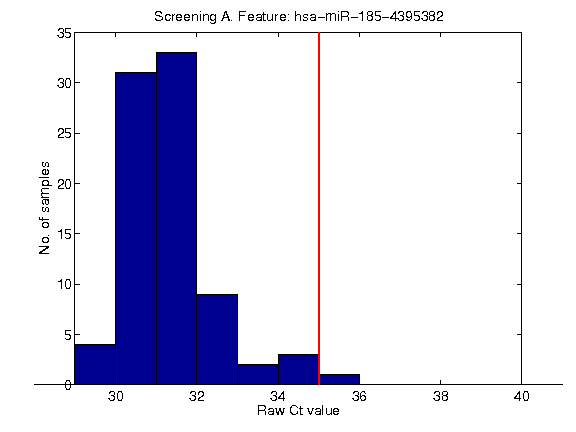 | 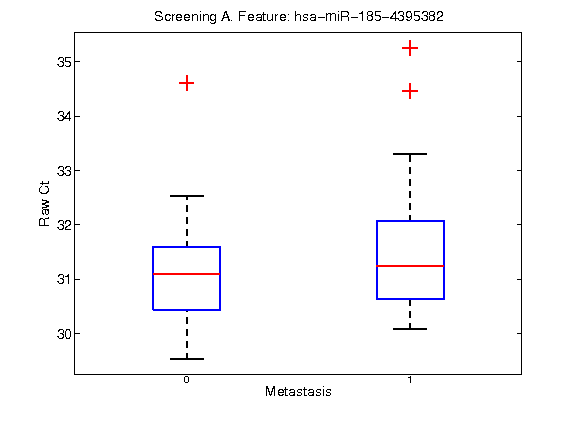 |
| miR-539 | 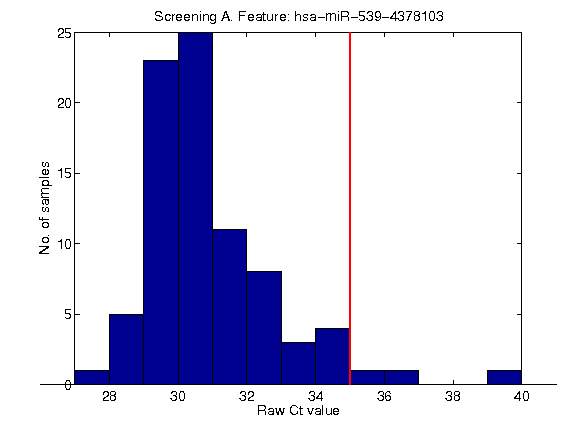 | 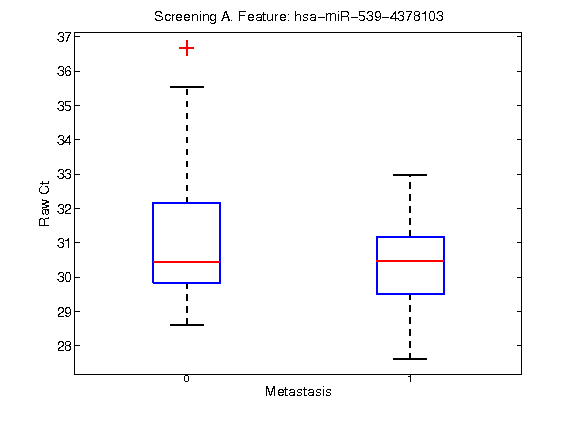 |
| miR-1296 | 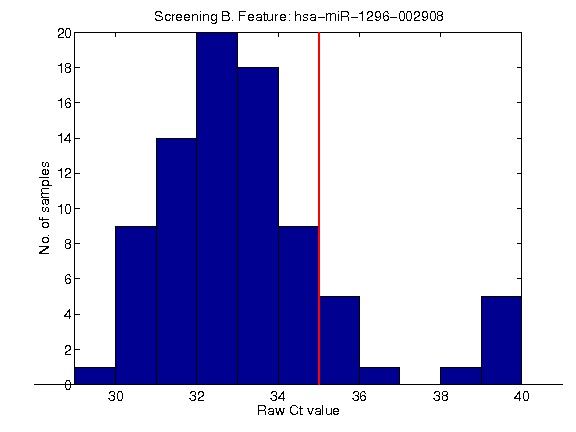 | 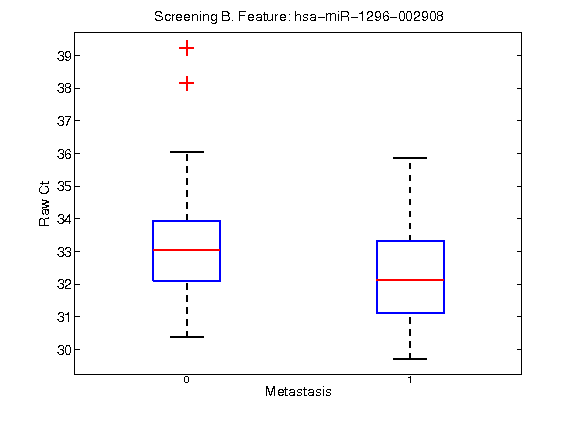 |
| miR-572 | 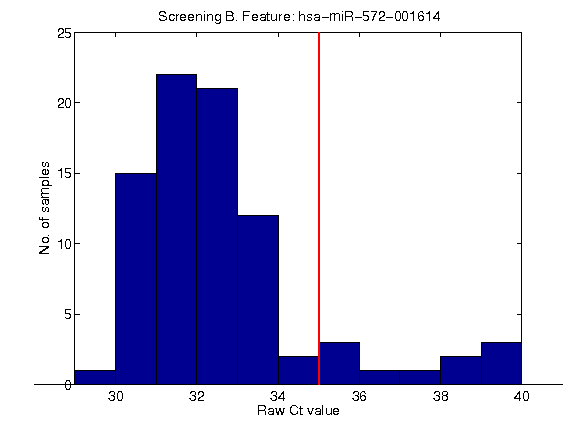 | 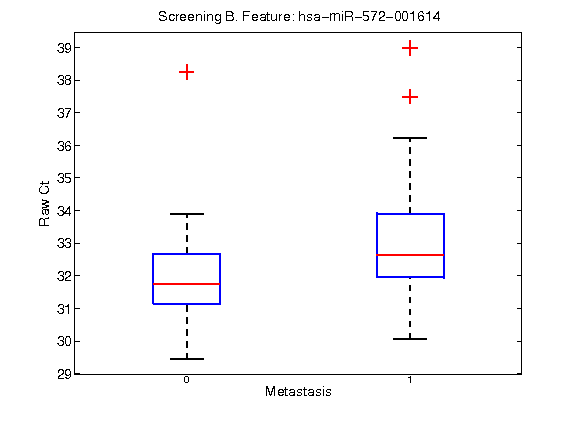 |
| miR-592 | 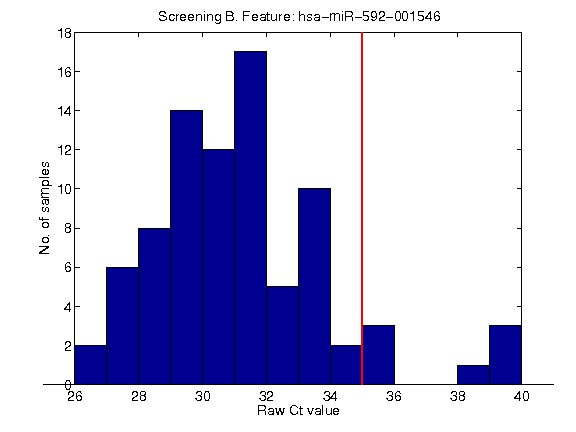 | 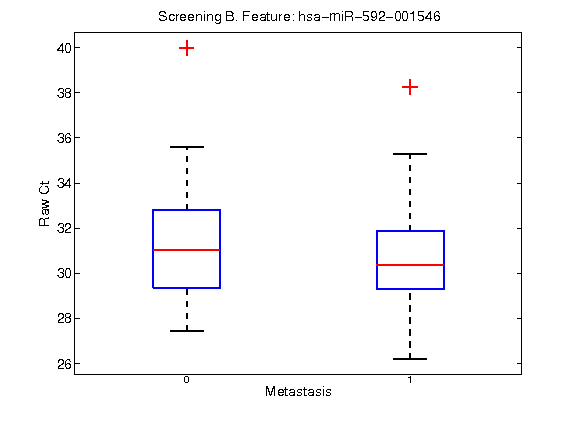 |
| miR-1300 | 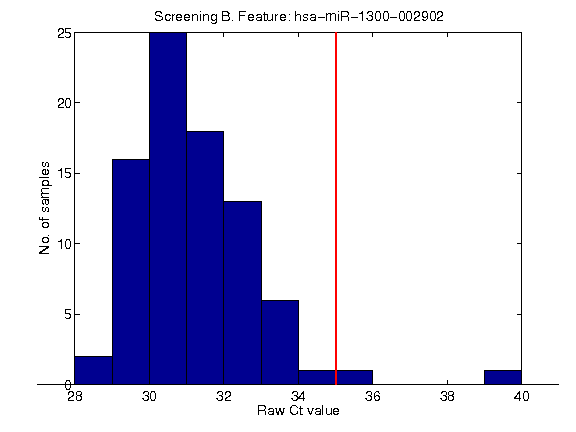 | 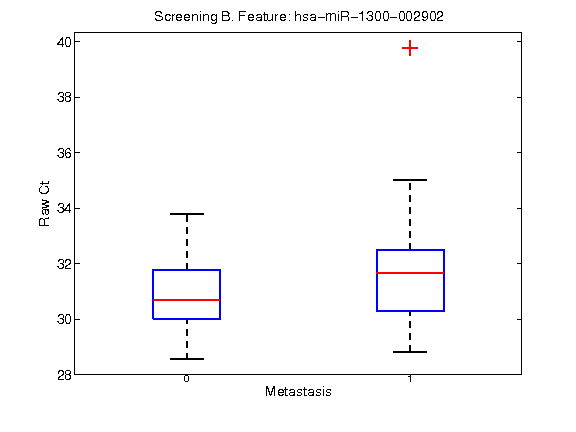 |
| miR-939 | 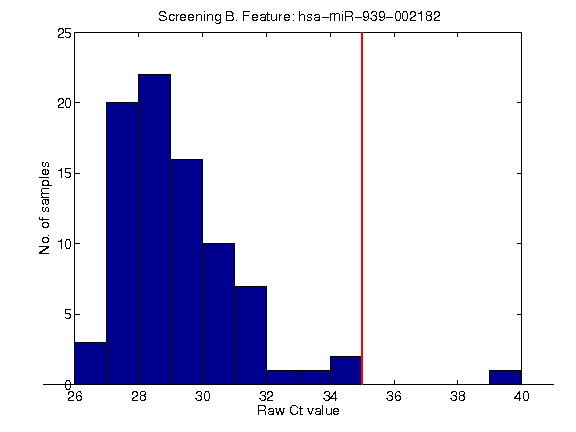 | 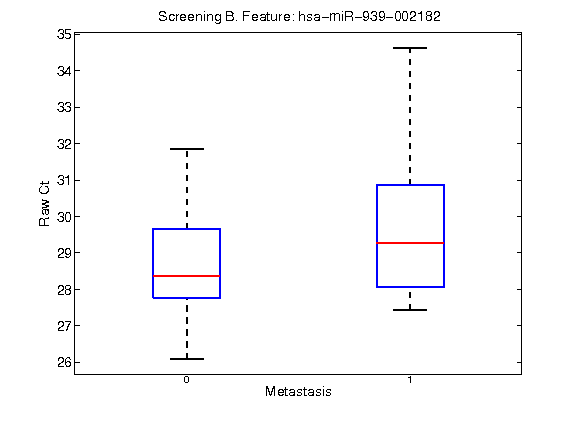 |

In the table below the percentages of samples with no amplification signal, when the threshold value for selected miRNAs was set at Ct=40 or at Ct=35, are presented. There were only few samples with no amplification signal. When the threshold value was set at 35, there was no significant difference for most of the miRNAs. Only for hsa-miR-1296 and hsa-miR-572 the percentage of samples with no amplification signal increased by no more than 15% for all samples, which is still a small part of all measurements.

| Type | miRNA name | % of samples with no amplification signal | | | | | |
| --- | --- | --- | --- | --- | --- | --- | --- |
|  |  | Ct threshold = 40 | | | Ct threshold = 35 | | |
|  |  | All | non-relapse | meta | All | non-relapse | meta |
| Signature | miR-135b | 1.20 | 2.22 | 0.00 | 1.20 | 2.22 | 0.00 |
|  | miR-185 | 0.00 | 0.00 | 0.00 | 1.20 | 0.00 | 2.63 |
|  | miR-539 | 1.20 | 2.22 | 0.00 | 3.61 | 6.67 | 0.00 |
|  | miR-1296 | 4.82 | 4.44 | 5.26 | 14.46 | 15.56 | 13.16 |
|  | miR-572 | 3.61 | 2.22 | 5.26 | 12.05 | 4.44 | 21.05 |
| MMR status | miR-592 | 2.41 | 4.44 | 0.00 | 8.43 | 8.89 | 7.89 |
| Cox reg. | miR-1300 | 0.00 | 0.00 | 0.00 | 2.41 | 0.00 | 5.26 |
|  | miR-939 | 1.20 | 2.22 | 0.00 | 1.20 | 2.22 | 0.00 |

**Normalization strategy**

The expression of valid control (‘normalising’) miRNA should not significantly vary between the analysed groups and should be present in all samples. Therefore, the miRNAs chosen as normalisers were to have the amplification signal in at least 95% of samples. For each candidate microRNA, expression stability value was calculated using NormFinder algorithm. NormFinder uses a mathematical model of miRNA expression that enables estimation of the overall variation of the candidate normalisers and of the variation between analysed groups defined by metastatic status (Andersen et al, 2004). Since expression level of miRNA in FFPE tissues spans across a broad Ct range (Ct 17-40), it is mandatory to include into normalisation panel miRNAs with different expression levels. Our normalisation procedure surmised inclusion of nine microRNAs with constant expression between metastatic and non-metastatic groups (Supplementary Table 1). These miRNAs belonged to one of the three groups based on average Ct value: low expression normalisers, with average Ct >30, medium expression normalisers, with average Ct >25-30, and high expression normalisers, with average Ct ≤25. For each group we chose three miRNAs with the lowest stability value inferred from NormFinder algorithm. The final group of nine normalisers was expanded by endogenous control miRNAs (U6 RNA, RNU44 and RNU48). Normalised expression of each miRNA was obtained with the ΔCt method, where ΔCt = Ct(target)miR – Ct(control)miR, and CtCONTROL is a geometric mean of Ct values measured for all estimated normalisers. Normalisation strategy was performed independently for experiments carried out with primer MegaPlex pools A and pool B (Life Technologies).

**Prognostic signature formulation process and multivariate prognostic models**

Multivariable logistic regression model was constructed to find a signature that describes the dependence between miRNA expression and recurrence status. For each patient Recurrence Score (RS) was estimated as prediction of logistic regression model. Given the large number of potential explanatory variables, regression model was limited to variables that were associated with p-values lower than 0.2 in the univariate analysis (Hosmer and Lemeshow, 2004). The number of model predictors was found using forward selection scheme, with manual tuning based on Bayesian Information Criterion (BIC), R2 and a p-value of F test indicating difference from a smaller model. Contribution of an individual predictor was measured using the Wald test. Optimal threshold for RS was found by maximizing positive predictive value (PPV) with constraint on NPV higher than 0.8. Additionally, regularised parameters of estimated model were obtained by using LASSO regression (Tibshirani, 1996). Prognostic abilities of obtained models were checked by internal leave-one-out cross-validation.

The clinical relevance of individual miRNAs was further tested in the Cox multivariate models that included three variables: miRNA expression (as -∆Ct), the T feature and the histological grade. The negative ∆Ct allowed for the intuitive interpretation of resulting HR values, e.g. for the cases where the high miRNA expression was associated with the high risk of relapse, the obtained HR value was greater than 1. The T feature was projected to a binary status (pT2 vs. pT3) and the grade was included with the values of 1, 2 or 3, respectively.

Andersen CL, Jensen JL, Ørntoft TF. Normalization of real-time quantitative reverse transcription-PCR data: a model-based variance estimation approach to identify genes suited for normalization, applied to bladder and colon cancer data sets. Cancer Res. 2004;64:5245-50.

Hosmer DW, Lemeshow S (2004) Applied Logistic Regression. Wiley.

Tibshirani R (1996) Regression shrinkage and selection via the lasso. J R Statist Soc B 58 (1): 267–288.
